# Rice transcription factors *OsNIGT2/3* regulate nitrogen acquisition by repressing *OsNRTs* and *OsAMTs* under high-nitrogen conditions

**DOI:** 10.3389/fpls.2025.1591808

**Published:** 2025-07-02

**Authors:** Hongyang Du, Xihan Cong, Xinmin Ruan, Yang Yang, Fuzhi Shi, Yanchang Luo, Zhixiang Luo

**Affiliations:** ^1^ Key Laboratory of Rice Genetic Breeding of Anhui Province, Rice Research Institute, Anhui Academy of Agricultural Sciences, Hefei, China; ^2^ College of Life Sciences, Zaozhuang University, Zaozhuang, China

**Keywords:** rice, OsNIGT2, OsNIGT3, OsNRTs, OsAMTs, NUE

## Abstract

**Introduction:**

Nitrogen is a crucial nutrient for crop growth, yet its utilization efficiency is generally low, leading to resource waste and serious environmental problems. Future agricultural sustainability requires improved crop NUE. In this study, we investigated the functions of the rice transcription factors *OsNIGT2* and *OsNIGT3* in nitrate uptake under high-nitrogen conditions.

**Methods:**

Hydroponic experiments and field tests were conducted to investigate the effects of *OsNIGT2* and *OsNIGT3* on physiological phenotypes and nitrogen use efficiency. Transcriptome analysis was used to explore the genome-wide transcriptional landscape of the two genes in response to nitrate availability. DNA affinity purification sequencing (DAP-seq) was employed to identify genomic sites bound by *OsNIGT2*, subsequently, yeast one-hybrid and transient expression assays verified the regulatory effects of *OsNIGT2* and *OsNIGT3* on key genes.

**Results:**

The double mutants of *OsNIGT2* and *OsNIGT3* presented significantly greater dry weights, nitrogen contents, total amino acid contents, and nitrate levels than the WT, whereas the single mutants presented no significant differences. These findings indicate the functional redundancy of *OsNIGT2* and *OsNIGT3* in regulating nitrogen uptake and assimilation. *OsNIGT2* and *OsNIGT3* act as transcriptional repressors, negatively regulating the expression of nitrogen absorption-related genes. Furthermore, DAP-seq identified potential targets bound by *OsNIGT2*, highlighting its role in the expression of several nitrogen and phosphorus utilization-related genes. Additionally, field tests shown that *OsNIGT2* and *OsNIGT3* knockout promotes both grain yield and NUE. This study provides potential genetic targets for improving yield and NUE in rice and other crops, laying a significant foundation for future crop improvement.

## Introduction

Nitrogen (N) is the most essential macronutrient for plant growth and serves as the primary limiting factor in most agricultural production. Over the past several decades, substantial amounts of nitrogen fertilizer have been applied to fields to achieve high crop yields. However, only 30–40% of the applied nitrogen is absorbed by crops, with a significant portion lost to the environment, leading to severe environmental pollution and ecological damage (Anas et al., 2020; Coskun et al., 2017). Additionally, the production of industrial ammonia relies on fossil fuels for hydrogen (H_2_) production, which consumes 2% of global energy and generates more than 400 million tons of CO_2_ emissions annually (Palys et al., 2021). Therefore, improving nitrogen use efficiency (NUE) is a critical issue that requires ongoing attention in agricultural production to maintain high yields while reducing pollution.

The primary forms of nitrogen utilized by plants in soil are nitrate (NO_3_^-^) and ammonium (NH_4_^+^) Nitrate transporters (NRTs) and ammonium transporters (AMTs) facilitate nutrient absorption in roots. Ammonium nitrogen (NH_4_^+^) is the preferred form for rice; however, due to nitrification around the rhizosphere, approximately 40% of the total nitrogen is absorbed as NO_3_^^-^^, and this form also plays a crucial role in increasing rice yield (Zhang and Chu, 2020). Vascular plants possess two types of nitrate transport systems, low-affinity transport systems (LATS) and high-affinity transport systems (HATS), which are adapted to environments with relatively high and low concentrations of NO_3_^-^, respectively. Nitrate transporters in higher plants are categorized into four families, namely, NPF (NRT1), NRT2, CLC, and SLAC/SLAH, with NRT1 and NRT2 playing the most significant roles (Krapp et al., 2014). Typically, NRT1s are considered to belong to the LATS, whereas NRT2s are considered to belong to the HATS. Notably, however, two NRT1 family members, *NRT1.1/CHL1* in *Arabidopsis* and *OsNRT1.1B*, its closest ortholog in rice, exhibit dual affinities for NO_3_^^-^^. In response to fluctuating ammonium levels, plants have also evolved high- and low-affinity ammonium transport systems (HATS and LATS) (Glass et al., 2002; Nazish et al., 2022).

The rice genome contains more than 80 *NRT1*, 4 *NRT2*, and 12 ammonium transporters (*AMTs*), which are divided into four subfamilies (OsAMT1-OsAMT4). The nitrate assimilation-related protein *OsNAR2.1* regulates the balance between ammonium and nitrate by interacting with multiple NRT2 family members (Islam, 2019; Nazish et al., 2022). Since the publication of the rice genome, numerous transporters and enzymes responsible for nitrogen absorption and assimilation have been identified through both forward and reverse genetics. Variations in certain genes, including *OsNRT1.1B*, *OsNPF6.1*, *OsNR2*, *OsNiR*, and *OsFd-GOGAT*, can influence nitrogen absorption, assimilation, and NUE. Additionally, several transcription factors (TFs) related to NUE, including *OsGRF4*, *OsNLP1*, *OsNLP4*, *OsNAC42*, *OsMYB305*, and *OsTCP19*, have been characterized in rice (Liu et al., 2021; Nazish et al., 2022).

Phosphorus (Pi) is another most abundant mineral nutrients utilized by plants, Pi transporters comprise different families, such as the phosphate transporter (PHT) family, the SYG1/Pho81/XPR1 (SPX) domain-containing protein family and the SULTR-like phosphorus distribution transporter (SPDT) family (Yang et al., 2024). *AtPHR1* and *OsPHR1/2/3* act as central regulators to activate the expression of phosphate starvation-induced (PSI) genes (Hu and Chu, 2019). Additionally, OsPHRs are required for mycorrhizal symbiosis, which facilitates root Pi uptake from the soil (Shi et al., 2021). The repressor proteins *OsSPX1/2/4*, which are degraded under Pi starvation, can interact with *OsPHR2* to block its cytoplasmic-nuclear shuttling activity under Pi-sufficient conditions, thereby inhibiting the expression of PSI genes. Thus, the OsSPX1/2/4-OsPHR2 module explains the mechanism of Pi starvation signal transduction (Shi et al., 2021). N and Pi coordinated utilization is vital for plants to achieve nutritional balance and optimal growth under a fluctuating nutritional environment. A synergistic effect of N and Pi co-fertilization on yield are well documented in many crops, and physiological observations that N act positively on Pi uptake and Pi starvation negatively on nitrate uptake and assimilation (Krouk & Kiba, 2020). Several molecular actors have been revealed controlling the molecular interaction between these two essential elements drafting a working model of N and Pi interactions (Krouk & Kiba, 2020).

Golden2-like (G2-like/GLK) TFs are members of the GARP family of Myb TFs and are known for their roles in chloroplast development, senescence, hormone response, and biotic and abiotic stress responses in plants (Chen et al., 2016; Safi et al., 2017). The NITRATE-INDUCIBLE GARP-TYPE TRANSCRIPTIONAL REPRESSOR1 (NIGT1)/HYPERSENSITIVE TO LOW Pi-ELICITED PRIMARY ROOT SHORTENING1 (HRS1) TF family, a subfamily of the G2-like TFs, is characterized by its rapid induction by nitrate and is involved in coordinating the absorption and utilization of nitrogen and phosphorus (Li et al., 2021). *AtNIGT1/HRS1* is hypersensitive to the shortening of primary roots induced by low phosphate and has six homologous genes, namely, *HHO1-6* (HRS1 homolog 1-6) (Liu et al., 2009). *HRS1*, *HHO1*, *HHO2*, and *HHO3* are strongly induced by nitrate; thus, they are also called *NIGT1.4*, *NIGT1.3*, *NIGT1.2*, and *NIGT1.1*, respectively. Further studies have revealed that the AtNIGT1/HRS1 subfamily integrates nitrate signaling and incorporates phosphorus starvation signals (Kiba et al., 2018; Medici et al., 2015; Wang et al., 2020b). These TFs can repress the nitrogen starvation response by inhibiting the high-affinity nitrate transporters *NRT2.1*, *NRT2.4*, and *NRT2.5* (Kiba et al., 2018; Safi et al., 2021). The high-affinity transport system (HATS) activity in the *hrs1;hho1;hho2;hho3* quadruple mutant is increased, accompanied by significant stimulation of growth (Safi et al., 2021).

*OsNIGT1* has been reported to belong to the HRS1/HHO family in rice, where it represses plant growth and mitigates phosphate starvation signaling to balance the trade-off in growth responses (Sawaki et al., 2013; Zhang et al., 2023). A total of four *OsNIGT1* homologs (i.e., *LOC_Os01g08160*, *LOC_Os03g55590*, *LOC_Os07g02800*, and *LOC_Os12g39640*) have also been identified in rice. *LOC_Os03g55590* and *LOC_Os07g02800* were induced by ammonium nitrate (Obertello et al., 2015). *OsHHO3* (*LOC_Os03g55590*) and *OsHHO4* (*LOC_Os07g02800*) were downregulated by N deficiency treatment, and predicted to function as key regulators in response to N deficiency (Ueda et al., 2020). It was reported that elimination of *OsHHO3* improves growth potential, NUE and yield under N-deficient conditions (Liu et al., 2023). In the present study, we found that *OsHHO3*, *OsHHO4* and *LOC_Os12g39640* were significantly induced by nitrate and are therefore designated *OsNIGT2* (*LOC_Os07g02800*), *OsNIGT3* (*LOC_Os03g55590*), and *OsNIGT4* (*LOC_Os12g39640*). *OsNIGT2* and *OsNIGT3* act as transcriptional suppressors that negatively regulate nitrate absorption in rice seedlings. Additionally, *OsNIGT2* and *OsNIGT3* have a broad impact on the transcriptome under N-sufficient conditions and can coordinate the expression of a subset of genes related to N utilization. Further studies have demonstrated that *OsNIGT2* and *OsNIGT3* repress N absorption by directly binding to the promoters of the NRT and AMT genes. We also provide evidence that *OsNIGT2* and *OsNIGT3* are potential regulators of Pi signaling and utilization. Our findings elucidate the regulatory network of the nitrate response in rice and suggest that *OsNIGT2* and *OsNIGT3* are promising candidates for improving NUE.

## Materials and methods

### Plant material and growth conditions

Nipponbare (*japonica*) was the wild-type rice variety selected for this study. Single *nigt2* and *nigt3* and double *nigt2/3* mutants were generated using the CRISPR-Cas9-mediated editing method (Xu et al., 2014). Appropriate sgRNA sequences were designed using the web-based tool CRISPR-P (http://crispr.hzau.edu.cn/CRISPR2/). The sgRNA construct was introduced into *Agrobacterium tumefaciens* strain EHA105, which was subsequently transferred into Nipponbare.

Hydroponic experiments were performed using modified Kimura B solution. Seeds were germinated on filter paper soaked in water for three days, after which uniform seedlings were transferred to a hydroponic box. Seedlings were grown in a growth chamber with a photoperiod of 16 hours light/8 hours dark and corresponding temperatures of 28°C/23°C, the illumination of the light source is 25,000 lux. The basal nutrient mixture consisted of the following macronutrients (in mM): 0.54 mM MgSO_4_·7H_2_O, 0.36 mM CaCl_2_·2H_2_O, 0.1 mM K_2_SO_4_, 0.18 mM KH_2_PO_4_, and 0.5 mM Na_2_SiO_3_·9H_2_O. The nutrient mixture also contained the following micronutrients (in μM): 9 μM MnCl_2_·4H_2_O, 20 μM H_3_BO_3_, 0.06 μM (NH_4_)_6_Mo_7_O_24_·4H_2_O, 0.76 μM ZnSO_4_·7H_2_O, 0.32 μM CuSO_4_·5H_2_O and 40 μM Fe-EDTA. The pH of the nutrient solution was maintained at 5.8. KNO_3_ was added to the basal nutrient mixture at the indicated concentrations for different treatments (0.2, 2, and 4 mM). The K^+^ concentration was adjusted with KCl to maintain consistency among the different conditions. Each nutrient mixture was replenished every two days.

### RNA extraction and RT–qPCR

Rice seedlings grown for two weeks in distilled water were treated with various compounds (4 mM KCl, KH_2_PO_4_, NH_4_Cl, and KNO_3_) for one hour, and the roots were collected at 0 and 1 hour. For time-course analyses of OsNIGT_2_ and OsNIGT_3_ expression in response to nitrate, the seedlings were grown in a nitrogen-free (0 mM KNO_3_) solution for two weeks. The experimental groups were treated with solution containing sufficient nitrogen (4 mM KNO_3_), whereas the control groups were treated with nitrogen-free (4 mM KCl) solution. The roots were harvested at 0, 0.5, 1, 2, 4, 8, and 12 hours after the treatments. Three biological replicates were performed for each analysis.

Total RNA was isolated from the roots using the RNAprep Pure Plant Kit (TIANGEN Biotechnology Co., Ltd.; code No. DP432), and cDNA was synthesized using the HiScript III First Strand cDNA Synthesis Kit (Vazyme Biotechnology Co., Ltd.; code No. R312). The qRT–PCR assays were performed using the Applied Biosystems QuantStudio 3 Real-Time PCR System (Life Technologies, USA) in conjunction with the ChamQ™ SYBR^®^ qPCR Master Mix (Vazyme Biotechnology Co., Ltd.; code No. Q331). Relative expression levels were normalized to those of *eIF4a* (AK073620) and calculated using the 2^^−ΔΔCt^ method. The primers used for RT–qPCR are listed in **Supplementary Table S3**.

### Subcellular localization

The full-length cDNA sequences encoding OsNIGT2 and OsNIGT3 were amplified from Nipponbare using the primers listed in **Supplementary Table S2**. The coding sequences were then subcloned and inserted into the pAN580 vector to construct *OsNIGT2-GFP* and *OsNIGT3-GFP* reporters, for transient transfection of rice protoplasts. A nuclear localization marker *OsWRKY29-RFP* was used. Rice protoplasts were isolated from the leaves of Nipponbare and transformed following a previously established protocol (Zhang et al., 2011). The *OsNIGT2-GFP* and *OsNIGT3-GFP* reporters were transformed into protoplasts and incubated in the dark for 16 to 20 hours at approximately 23°C, after which they were observed using an LSM780 Exciter confocal laser scanning microscope (Zeiss, Germany).

### Promoter–GUS analyses

A 1892 bp promoter region of OsNIGT2 and a 2119 bp promoter region of OsNIGT3 were amplified from Nipponbare using the primers described in **Supplementary Table S2** and subsequently subcloned and inserted into pCAMBIA-1381Z to generate *OsNIGT2pro:GUS* and *OsNIGT3pro:GUS*. The construct was introduced into *Agrobacterium tumefaciens* strain EHA105, which was then transferred into Nipponbare. For GUS staining, seedlings of *OsNIGT2pro:GUS* and *OsNIGT3pro:GUS* transgenic rice were grown for 2 weeks with distilled water and subsequently treated with various compounds (4 mM KCl, KH_2_PO_4_, NH_4_Cl, KNO_3_, and NH_4_NO_3_) for 3 hours, and roots were collected at 0 and 1 hour after the end of the treatment period. Samples were soaked overnight at 37°C in darkness in GUS assay solution containing 0.5 mg/ml 5-bromo-4-chloro-3-indolyl glucuronide, 0.1% Triton X-100, 1 mM K_3_[Fe(CN)_6_], 1 mM K_4_[Fe(CN)_6_], 10 mM EDTA and 50 mM sodium phosphate buffer. The stained plants were cleared via incubation in a 75% ethanol series and observed via light microscopy.

### Total N, nitrate, and amino acid concentration assays

In the hydroponic experiment, the seedlings were grown to the six-leaf stage (V6). Shoots and roots were harvested, subjected to enzyme denaturation at 105°C for 10 min, and then dried at 70°C for 3 days. The N content in the shoots and roots was estimated via the Kjeldahl method. In addition, samples of leaves and roots were harvested for measurements of total amino acid and nitrate (NO_3_^¯^) concentrations using kits (AA-1-W and ZXTD-1-G, respectively) obtained from Suzhou Comin Biotechnology Co., Ltd. All analyses were performed in six biological replicates.

### Chlorate sensitivity assay

Seedlings of *nigt2/3* double mutants and Nipponbare were first cultured in nutrient solution containing 2 mM KNO_3_ for 4 days after germination. The seedlings were subsequently treated with 2.5 mM KClO_3_ for 4 days and allowed to recover in nutrient solution (2 mM KNO_3_) for 4 days. The experiment was carried out in four replications, each containing 96 plants per genotype.

### Transcriptome sequencing and analysis

*Nipponbare* and the *nigt2/3-ko1* double mutant were cultured in solutions with varying nitrate concentrations (0.2 mM and 4 mM). Roots from 3-week-old seedlings were sampled for transcriptome sequencing. The samples were prepared for sequencing using the Illumina TruSeq™ RNA Sample Preparation Kit, and sequencing was conducted on an Illumina HiSeq 2500 instrument by Biozeron Biotechnology Co., Ltd. Following RNA-seq, the reads were aligned to the reference genome using HISAT2, and fragments per kilobase of transcript per million mapped reads (FPKM) values were calculated using featureCounts. Differentially expressed genes (DEGs) with a false discovery rate (FDR) of less than 0.05 and a fold change greater than 2 were identified using edgeR. Gene Ontology (GO) annotation was performed using Goatools (available at https://github.com/tanghaibao/GOatools). Three biological replicates were used for all analyses.

### DAP-seq and analysis

DAP-seq was performed as previously described (Bartlett et al., 2017), with some modifications. Genomic DNA (gDNA) was extracted from Nipponbare roots and sonicated for 5 min using a Bioruptor Pico (Diangenode, Belgium). The fragmented gDNA was subsequently ligated with a truncated NEXTflexTM barcode adapter to create the DNA library. The OsNIGT2 CDS was cloned and inserted into the pFN19K HaloTag^®^ T7 SP6 Flexi^®^ Vector. HALO-TF fusion proteins were expressed in an *in vitro* wheat germ system (TNT^®^T7 Coupled Wheat Germ Extract System), immobilized on Magne HALO-Tag beads, washed, and incubated with the DNA library for 1 hour with horizontal rotation at 25°C. The beads were washed again, and then the DNA was eluted and amplified with indexed TruSeq primers (NEXTflex Rapid DNA-seq Kit, Bioo Scientific, code No. NOVA-5144-08). GFP was used as a mock control. Sequencing was performed on an Illumina HiSeq™ 2500 system.

The raw sequencing reads from the DAP-seq experiments were aligned to the *Nipponbare* reference genome using the Burrows–Wheeler Aligner (BWA). Peak calling was conducted using Model-based Analysis of ChIP-seq (MACS2). Peaks were deemed significant and considered for further analysis if they met the criteria of p < 10^-3 and fold change > 2 with high confidence in both biological replicates. Significant peaks located within the promoter regions or 5’ UTRs of protein-coding genes were hypothesized to potentially bind to OsNIGT2. To identify binding motifs within these significant OsNIGT2-binding regions, a 400 bp sequence surrounding the peak summit was extracted and analyzed using the online tool MEME-ChIP (available at https://meme-suite.org/meme/tools/meme-chip).

### Y1H assay

The Y1H assay was conducted with the Matchmaker Gold Yeast One-Hybrid System (Clontech) in accordance with the manufacturer’s protocol. The promoter regions of seven target genes (*OsNRT1.1A, OsNRT1.1B, OsNRT2.2, OsAMT1.2, OsAMT1.3, OsAMT2.1*, and *OsNIGT1)* were amplified from Nipponbare using the primers listed in **Supplementary Table S2**. The CDSs of OsNIGT2 and OsNIGT3 were subsequently cloned and inserted into the pGADT7 vector to generate the effector constructs pGADT7-NIGT2 and pGADT7-NIGT3. The 400 bp sequences flanking the B-box motif of the promoters were cloned and inserted into the pAbAi vector, linearized by BstBI digestion, and transformed into the Y1HGold strain to produce reporter strains. These strains were subsequently transformed with either pGADT7 or pGADT7-NIGT2/pGADT7-NIGT3. The transformants were then spread on SD/-Leu media supplemented with 250 ng/mL aureobasidin A.

### Transient expression assays in *Nicotiana benthamiana* leaves

To investigate the transcriptional inhibitory activity of *OsNIGT2* and *OsNIGT3*, the corresponding CDSs were cloned and inserted into the vector pMDC83-BD, which contains the DNA binding domain of GAL4, to generate the effector constructs pMDC83-BD-NIGT2 and pMDC83-BD-NIGT3. The reporter vector was constructed with a 35S promoter driving the expression of UAS-LUC. The effector, reporter, and reference constructs were transiently coexpressed in the leaves of *Nicotiana benthamiana* via *Agrobacterium* GV3101 infiltration, following previously established protocols (Sparkes et al., 2006). After a 24-hour incubation in the dark and an additional 24 hours in light, the reporter and reference values were quantified using the Dual Luciferase Reporter Gene Assay Kit (Yeasen Biotechnology Co., Ltd.; code No. 11402ES60) with the Agilent BioTek Cytation5 system.

To generate reporter constructs, the promoters of four genes (OsNRT1.1A, OsNRT1.1B, OsNRT2.2, and OsAMT2.1) were cloned and inserted into the vector pGreenII0800-LUC. The CDSs of OsNIGT2 and OsNIGT3 were also inserted into the vector pMDC83, yielding the effector constructs pMDC83-NIGT2 and pMDC83-NIGT3. The reporter and effector constructs were then transiently coexpressed in the leaves of *Nicotiana benthamiana*. Following incubation, the reporter and reference values were measured as previously described.

### Field tests of rice

Single *nigt2* and *nigt3*, double *nigt2/3* mutants and WT were used for field tests, which performed in the pool fields under natural growth condition at Changfeng biological breeding research and test base (Hefei, Anhui Province). The nutritional composition of the soils in each pool were tested. The alkali-hydrolyzale nitrogen, available potassium and phosphorus of the low N (LN) experimental field were 73.3, 177.5 and 45.0 mg/kg. The alkali-hydrolyzale nitrogen, available potassium and phosphorus of the high N (HN) experimental field were 71.7, 179.4 and 40.3 mg/kg. Urea (N content 46%) was used as the N source with none for LN and 240 kg N/hm^2^ for HN. The plants were transplanted in 16 rows × 9 plants for each plot, and the spacing between rice plants was 26.4 cm × 16.7 cm. Superphosphate (P_2_O_5_ content 12%) was used as phosphorus fertilizer (75 kg P/hm^2^) and muriate of potash (K_2_O content 60%) was used as potassium fertilizer (150 kg K/hm^2^) in the paddy field. Four replicates were used.

After ripening, the middle 5 plants in the middle row were selected to investigate the agronomic traits, including plant height, tiller number, thousand kernel weight and grain yield. The agronomic NUE (aNUE) were calculated according to the following formula:

aNUE= (yield in HN - yield in LN)/nitrogen fertilizer rate.

## Results

### *OsNIGT2* and *OsNIGT3* encode nitrate-induced transcription repressors

**Figure 1:**
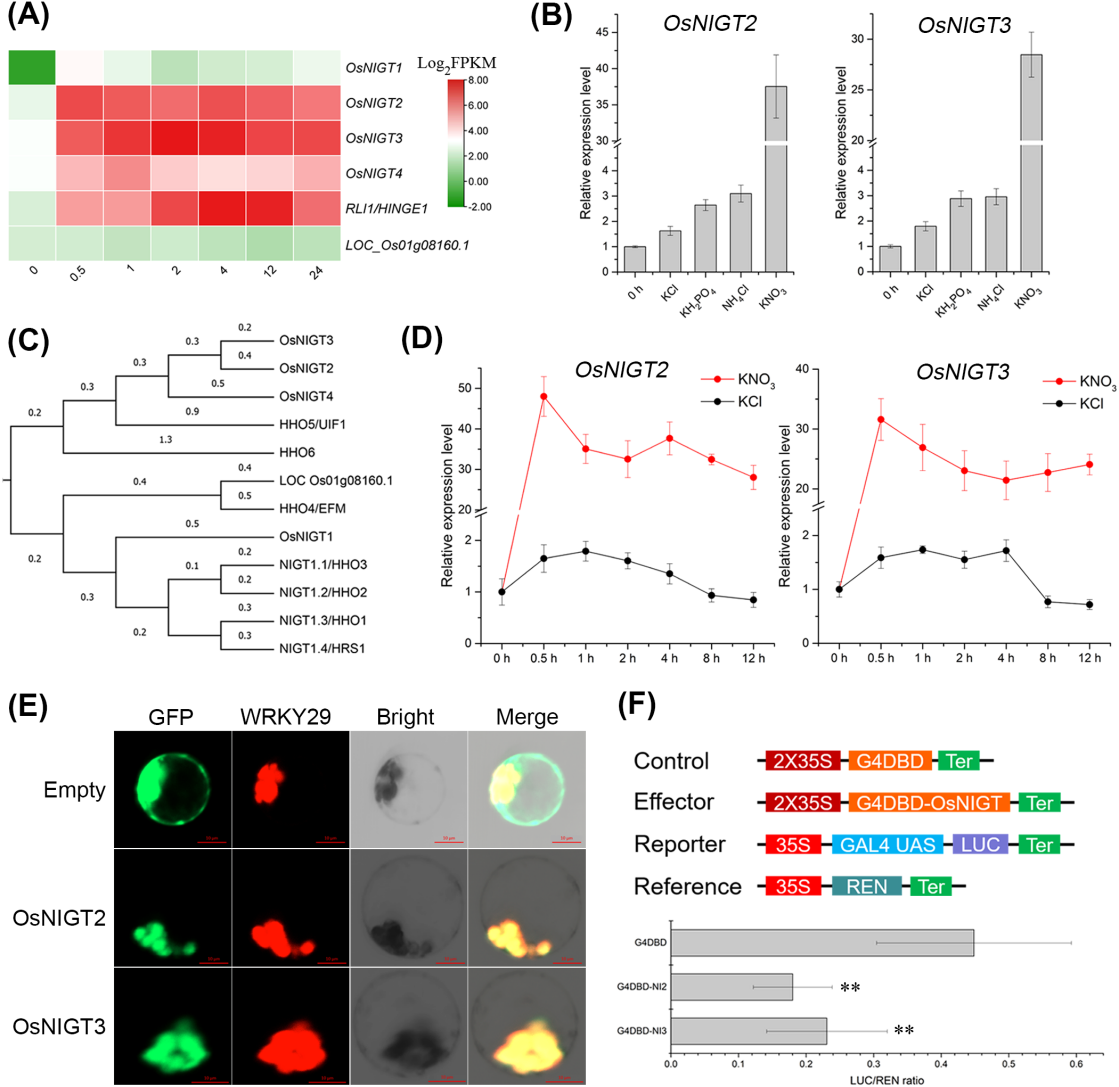
OsNIGT2 and OsNIGT3 are transcriptional repressors that are strongly induced by nitrate. **(A)** Heatmap of G2-like transcription factors expression under NH_4_NO_3_ treatment, the number represents the processing time (hour). **(B)** Nitrate-specific induction of *OsNIGT2* and *OsNIGT3* expression. **(C)** A phylogenetic tree of the G2-like transcription factor subfamily constructed by ClustalW alignment and the maximum likelihood method. **(D)** Time-course analyses of *OsNIGT2* and *OsNIGT3* expression levels in response to nitrate. **(E)** The OsNIGT2-GFP and OsNIGT3-GFP signals colocalize with the cell nucleus marker OsWRKY29 (fused with RFP), bars = 10 μm. **(F)** OsNIGT2 and OsNIGT3 transcriptional repression assay. Asterisks indicate significant differences of LUC/REN ratio between the Empty and OsNIGT2/NIGT3 according to two-tailed Student’s t tests: **,P<0.01.

To investigate the molecular mechanisms underlying the response to external nitrogen, we examined the time course changes in the transcriptome of rice roots following NH^4^NO_3_ resupply after nitrogen deficiency. Among the 79 transcription factors (TFs) identified as differentially regulated, seven belong to the G2-like family, with five exhibiting highly significant NH^4^NO_3_-induction of expression (**Figure 1A**; **Supplementary method and Table S1**). RLI1 is closely related to PHR proteins and modulates nitrate-induced phosphate starvation signaling (Guo et al., 2022; Zhang et al., 2021). The remaining four TFs belong to the NIGT subfamily; in addition to *OsNIGT1*, the other three are designated *OsNIGT2* (LOC_Os07g02800), *OsNIGT3* (LOC_Os03g55590) and *OsNIGT4* (LOC_Os12g39640), all these three contain conserved HGD (hydrophobic and globular domain) and GARP motif (**Supplementary Figure S1**). Interestingly, *OsNIGT2*, *OsNIGT3*, and *OsNIGT4* are phylogenetically more similar to *AtHHO5* and *AtHHO6* than to the TFs in the NIGT1 clade (**Figure 1C**). These findings suggest that similar yet distinct proteins may play significant roles in the response to nitrogen supply and deficiency. Considering their redundancy, *OsNIGT2* and *OsNIGT3* were selected for further study.

**Figure 2:**
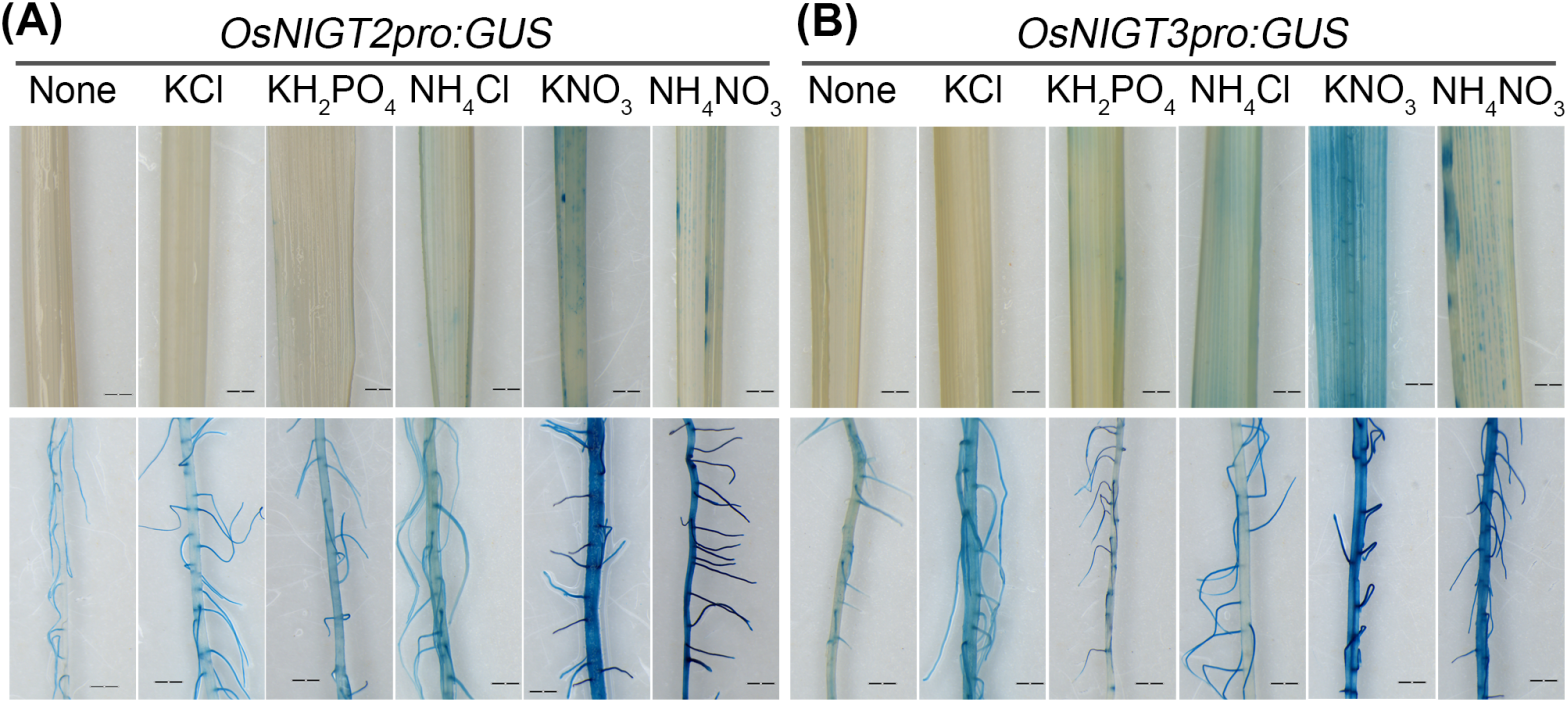
Spatial expression patterns of *OsNIGT2* and *OsNIGT3*. **(A)** Spatial patterns of *OsNIGT2* expression as detected by GUS staining in *OsNIGT2pro:GUS*. **(B)** Spatial patterns of *OsNIGT3* expression as detected by GUS staining in *OsNIGT3pro:GUS*. Two-week-old seedlings were incubated with various compounds (4 mM KCl, KH_2_PO_4_, NH_4_Cl, KNO_3_, and NH_4_NO_3_) for 3 hours before GUS staining. Bar = 200 mm.

To investigate the specificity of the response of *OsNIGT2/3/4* to various nitrogen sources, we monitored the expression in the roots of rice treated with different inorganic nitrogen compounds following nitrogen deficiency. The expression of *OsNIGT2/3/4* was significantly induced by NO_3_^¯^ (**Figure 1B**; **Supplementary Figure S2a**). β-Glucuronidase staining revealed the detailed expression pattern of *OsNIGT2pro:GUS* and *OsNIGT3pro:GUS* under various treatments (**Figure 2**). *OsNIGT2* and *OsNIGT3* are expressed primarily in root hairs, and they are expressed at low levels even in the absence of external mineral elements. According to the staining results, KCl, KH_2_PO^4^, and NH^4^Cl did not induce GUS expression. In contrast, KNO_3_ and NH^4^NO_3_ did induce GUS expression, which was also greater in leaves. Time course analysis revealed that *OsNIGT2* and *OsNIGT3* were induced within 0.5 hours of nitrate treatment, followed by a decrease in expression upon further nitrate exposure (**Figure 1D**), suggesting that OsNIGT2 and OsNIGT3 are important regulators involved in the primary nitrate response.

To determine the subcellular localization of *OsNIGT2* and *OsNIGT3*, we cotransformed the *OsNIGT2-GFP* and *OsNIGT3-GFP* fusion proteins into rice protoplast cells along with a nuclear localization marker, *OsWRKY29-RFP*. The GFP fluorescence signal indicated that both the *OsNIGT2-GFP* and the *OsNIGT3-GFP* fusion proteins were predominantly localized in the nucleus (**Figure 1E**). To assess the transcriptional activity of *OsNIGT2* and *OsNIGT3*, we conducted a dual luciferase reporter assay. Effector and reporter plasmids were cotransformed into tobacco leaves, and REN luciferase was used as an internal reference (**Figure 1F**). The LUC luciferase activity was significantly repressed in the experimental case, revealing that *OsNIGT2* and *OsNIGT3* act as transcriptional suppressors to directly downregulate gene expression.

### *OsNIGT2* and *OsNIGT3* knockout resulted in increased N accumulation

**Figure 3:**
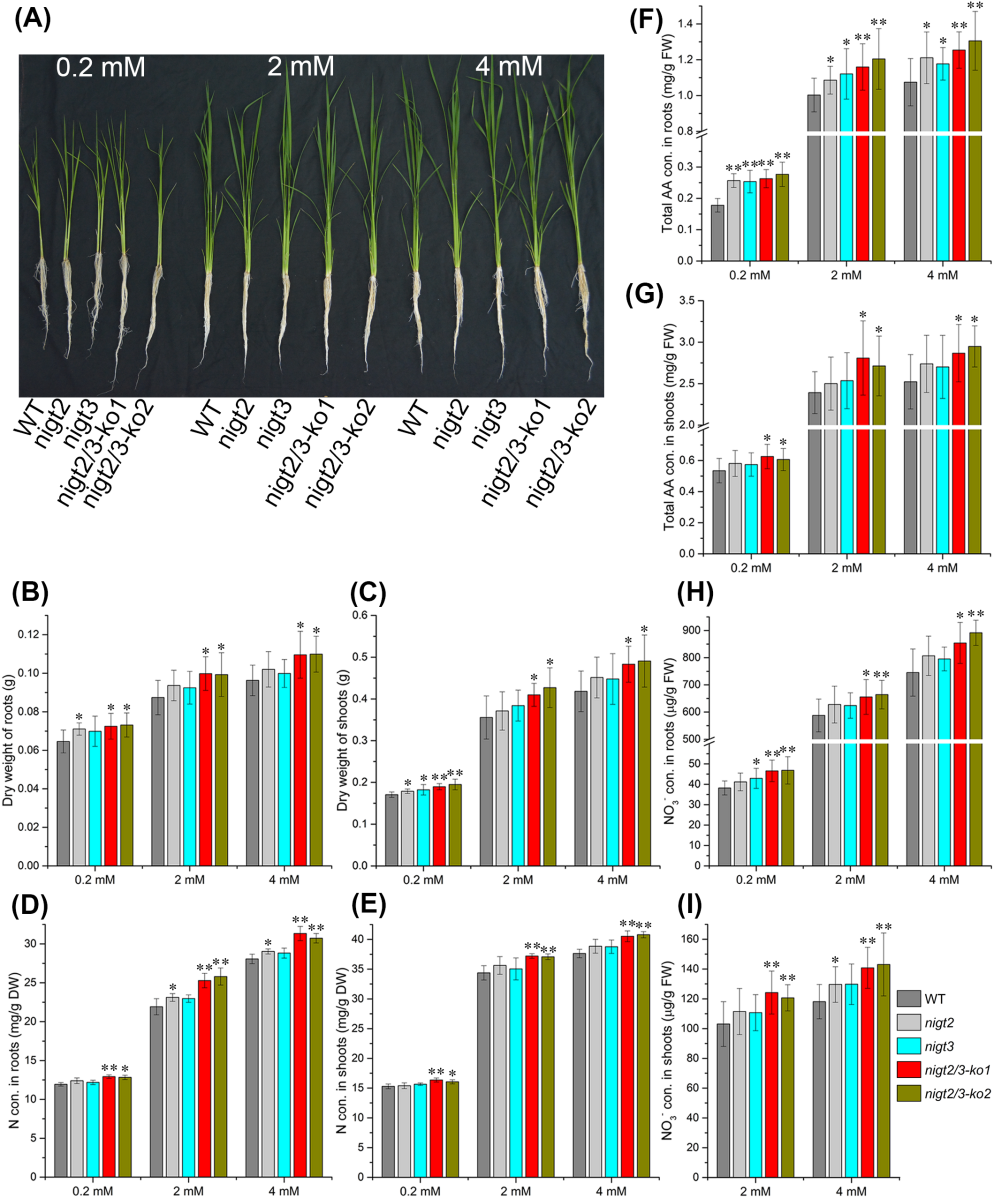
OsNIGT2 and OsNIGT3 knockout increased N accumulation in hydroponic culture. **(A)** Growth morphology of Nipponbare (WT), *nigt2*, *nigt3*, and *nigt2/3* (two lines) grown in hydroponic culture. **(B, C)** Dry weights of shoots and roots. **(D, E)** Total N concentrations in shoots and roots. **(F, G)** Total amino acid concentrations in leaves and roots. **(H, I)** NO_3_^¯^ concentrations in leaves and roots. Con, concentration; DW, dry weight. Asterisks indicate significant differences between the WT and *nigt2* or *nigt3* single mutants or double *nigt2/3* mutant according to two-tailed Student’s t tests: *, P < 0.05; **, P < 0.01. Detailed data were shown in **Supplementary Table S4**.

To further confirm the roles of *OsNIGT2* and *OsNIGT3* in rice growth and development, we generated *OsNIGT2/3* single and double mutant lines using CRISPR/Cas9 technology (**Supplementary Figure S2b**). T2 seedlings were assessed for phenotypes in hydroponic cultures under three nitrate conditions (0.2, 2 and 4 mM). Compared with the wild type (WT), the single *nigt2* and *nigt3* mutants presented no visible differences in terms of plant height or root length; however, the dry weights of the shoots and roots under the 0.2 mM nitrate condition were higher than that of the WT (**Figures 3A–C**). The nitrogen concentrations in the tissues also did not differ between the single mutants and the WT, except that the root nitrogen concentration under the 2 mM nitrate condition was increased in *nigt2* (**Figures 3D, E**). The total amino acid concentrations were higher in the roots of single mutants, whereas the concentrations in the shoots did not differ (**Figures 3F, G**). Additionally, the NO_3_^-^ concentrations did not differ between the single mutants and WT, except for increased concentrations in the roots of *nigt3* and in the shoots of *nigt2* at 0.2 mM and 2 mM, respectively (**Figures 3H, I**).

**Figure 4:**
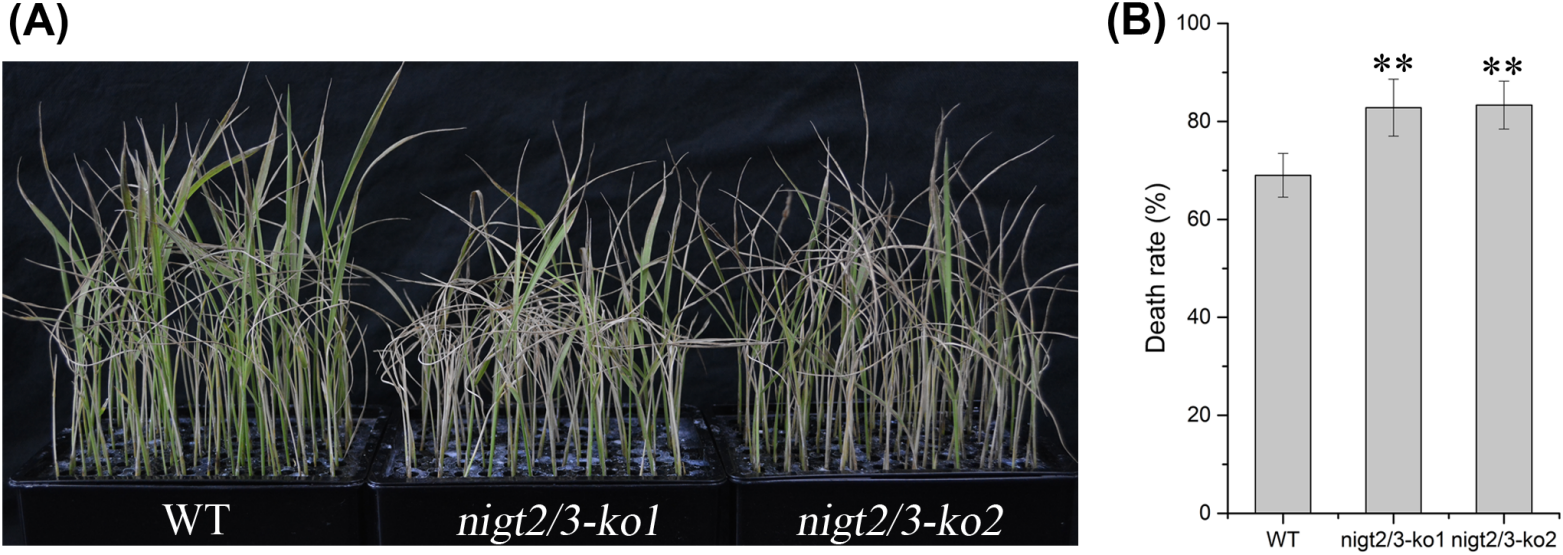
Chlorate sensitivity assay in Nipponbare (WT) and *nigt2/3* double mutants. **(A)** Growth morphology of Nipponbare (WT) *nigt2/3* double mutants grown in hydroponic culture. **(B)** Chlorate sensitivity was calculated from the mortality rate of plants poisoned with chlorate. Asterisks indicate significant differences of death rate between the WT and double nigt2/3 mutant according to two-tailed Student’s t tests: **,P<0.01.

The *nigt2/3* double mutants also exhibited no visible differences in plant height or root length under high nitrate conditions (2 and 4 mM), but the root length of the double mutants increased at 0.2 mM (**Figure 3A**). Furthermore, the dry weights and nitrogen, total amino acid, and NO_3_^^-^^ concentrations of the shoots and roots of the double mutants were significantly higher than that of WT under all three nitrate conditions (**Figures 3B–I**). Combined with the phenotypes of the single mutants, these observations suggest that *OsNIGT2* and *OsNIGT3* are functionally redundant in regulating nitrogen uptake and assimilation. To confirm these physiological findings, we investigated the role of *OsNIGT2/3* in regulating nitrate absorption through a chlorate sensitivity assay. We found that the double mutants presented significantly greater chlorate sensitivity than did the WT (**Figures 4A, B**), demonstrating that *OsNIGT2/3* negatively modulates nitrate uptake.

### *OsNIGT2* and *OsNIGT3* coordinate N utilization-related gene expression under nitrate sufficiency

**Figure 5:**
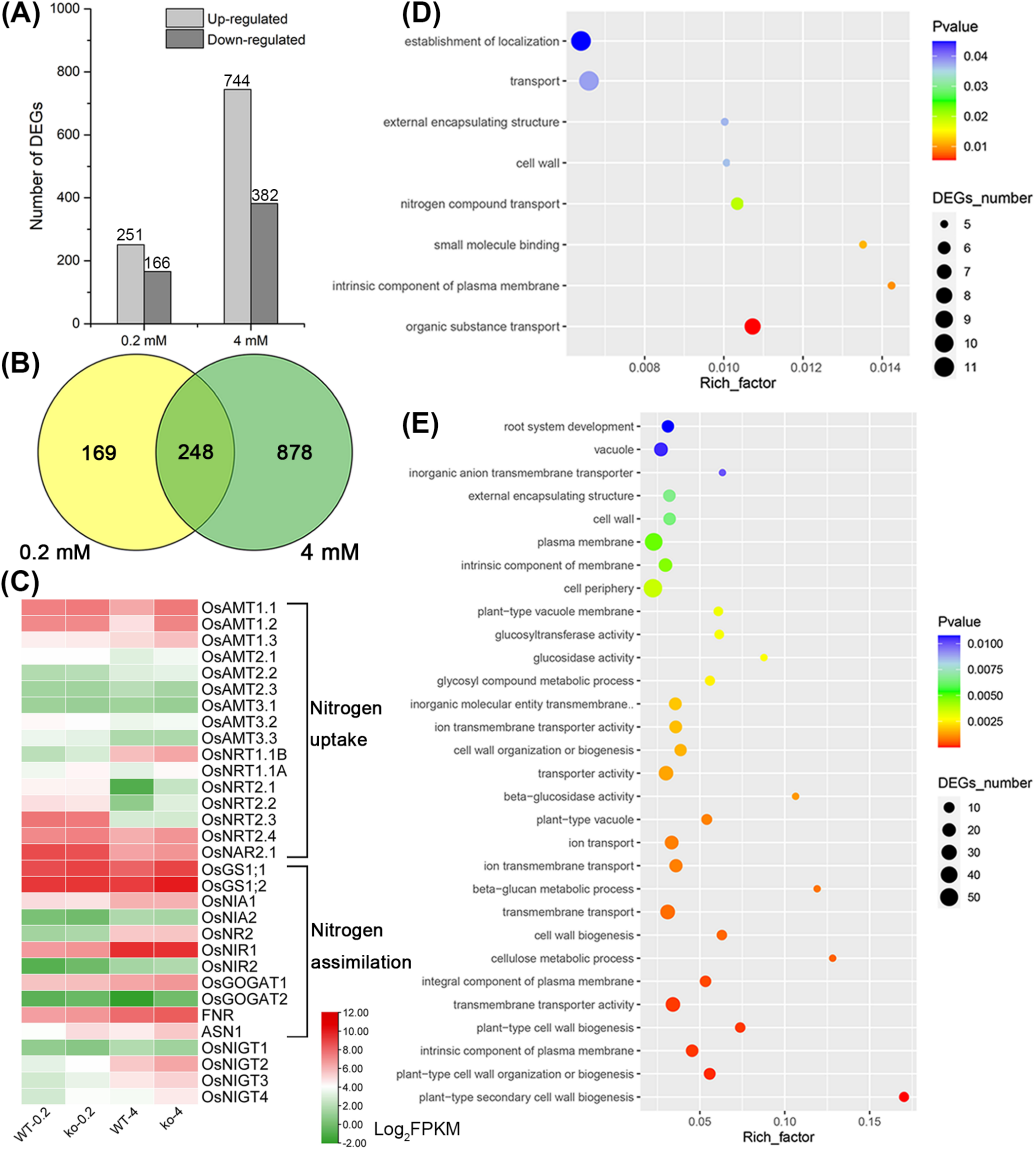
Expression profiling analysis of Nipponbare and *nigt2/3-ko1* double mutant seedlings under different nitrate concentrations. **(A)** Number of upregulated and downregulated genes in ko vs. WT under LN (0.2 mM KNO_3_) and HN (4 mM KNO_3_) conditions. **(B)** Venn diagram analysis of the common and specific genes unique to and shared between the different pairwise comparisons. **(C)** Heatmap of nitrogen uptake and assimilation genes under LN and HN conditions. **(D)** Gene Ontology (GO) classification of DEGs from *nigt2/3-ko1* vs. WT under LN conditions. **(E)** GO classification of DEGs from *nigt2/3-ko1* vs. WT plants under HN conditions.

To investigate the molecular mechanisms by which *OsNIGT2* and *OsNIGT3* regulate nitrogen uptake and assimilation, we explored the genome-wide transcriptional landscape in response to nitrate availability by profiling the transcripts of *nigt2/3-ko1* double mutant and WT plants at two nitrate concentrations (0.2 mM and 4 mM). We identified 417 differentially expressed genes (DEGs) under 0.2 mM nitrate, with 251 upregulated and 166 downregulated in the double mutant compared to WT; whereas under 4 mM nitrate, there were 1126 DEGs, including 744 upregulated and 382 downregulated (**Figures 5A, B**, **Supplementary Data S1**). The observation that the number of DEGs at 4 mM was significantly greater than that at 0.2 mM indicates that *OsNIGT2* and *OsNIGT3* function primarily at high NO_3_^-^ levels. This finding aligns with the expression patterns of *OsNIGT2* and *OsNIGT3*, which exhibit low expression under low nitrate conditions and high expression under nitrate-rich conditions.

We further classified the DEGs using Gene Ontology (GO) analysis. As shown in **Figures 5D, E**, the DEGs were significantly enriched among GO terms related to “transport activity” and “cell wall biogenesis”, indicating that OsNIGT2/3 may coordinately regulate key genes involved in N transport and root development. Interestingly, OsNIGT2/3 appeared to negatively regulate the expression of high-affinity nitrate/ammonium transporters, such as *OsNRT2.1*, *OsNRT2.2*, *OsAMT1.1* and *OsAMT1.2*, and dual-affinity nitrate transporters, such as *OsNTR1.1A* and *OsNRT1.1B*, at 4 mM (**Figure 5C**). In addition, several key genes involved in N assimilation, such as *OsGS1;1* (1.78-fold), *OsGS1;2* (1.85-fold), *OsGOGAT2* (3.47-fold), *FNR* (1.33-fold), and *ASN1* (1.91-fold), were upregulated in the *nigt2/3-ko1* double mutant. Taken together, these results suggest that OsNIGT2/3 are pivotal regulators that coordinate N metabolism under nitrate-sufficient conditions. The expression of *OsNIGT2/3/4* also increased in the double mutant, although none of these genes were upregulated by more than twofold (**Figure 5C**; **Supplementary Figure S3**), suggesting that a feedback inhibition mechanism exists in the OsNIGT clade. The expression patterns of the genes involved in N uptake and assimilation were verified by RT–qPCR, the results of which were largely consistent with the RNA-seq data (**Supplementary Figure S3**).

### DNA affinity purification sequencing identifies genomic sites that are bound by *OsNIGT2*

**Figure 6:**
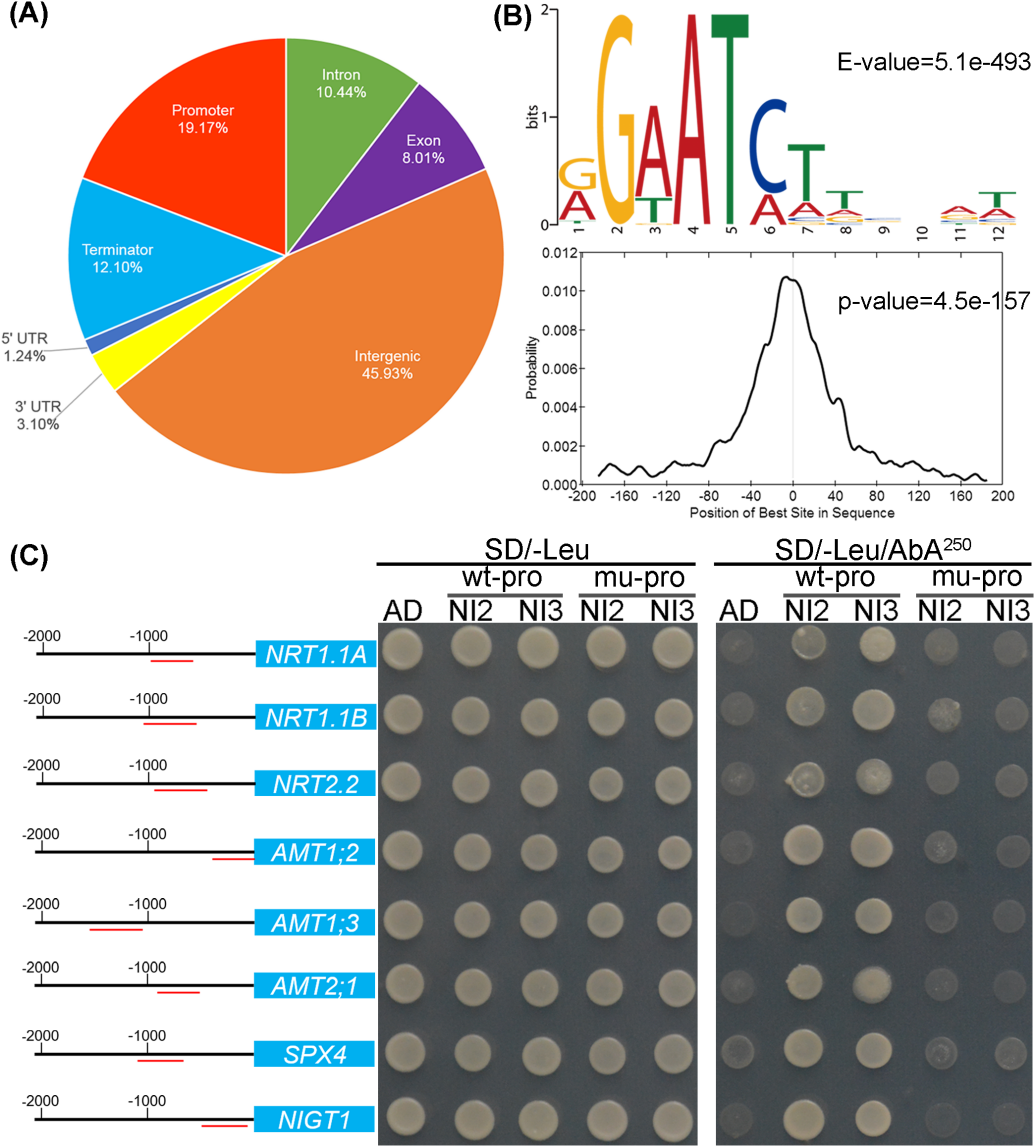
Genome-wide binding profiles of OsNIGT2 from the DAP-seq analysis. **(A)** Distribution of OsNIGT2-binding regions in the rice genome. Promoter region, -2 kb to the transcription start site (TSS); terminator, +2 kb to the transcription termination site (TTS); intergenic region, 2 kb upstream of the TSS or 2 kb downstream of the TTS. **(B)** OsNIGT2-binding motif identified by MEME-ChIP in 400 bp flanking sequences around the genic peak summits and density plot of this motif around peak summits. **(C)** Y1H assay of OsNIGT2 and OsNIGT3 interactions with potential promoters. NI2, pGADT7-NIGT2; NI3, pGADT7-NIGT3; wt-pro represents the original promoter sequence; mu-pro represents a mutation in the B-box of the promoter (GAATATTC → tAcgATTC or GAATC → tAcgC).

DNA affinity purification sequencing (DAP-seq) was employed to identify genomic sites bound by *OsNIGT2*, specifically, to identify cis-regulatory elements (CREs) directly targeted by the OsNIGT2 protein. A total of 7,354 peaks were identified in comparison with the negative control (GFP). Among all the detected peaks, 45.93% were located in intergenic regions, whereas 19.17% were found in core promoter regions (**Figure 6A**). The peaks situated in the promoter or 5’ untranslated region (5’ UTR) of protein-coding genes were subjected to further analysis, resulting in 1,254 peaks distributed across 1,201 genes (**Supplementary Data S2**). The *de novo* discovery of enriched motifs in the binding sites of the promoter and 5’ UTR regions identified the B-box (GAATC/AT) as the top-scoring motif (E-value = 5.1e-904; **Figure 6B**). This B-box was found to be identical to those of OsNIGT1 and the binding sequences of *Arabidopsis* NIGT1 (GAATATTC and GAATC).

Among the 1,201 potential genes regulated by *OsNIGT2*, 835 were annotated, and the only GO term found to be significantly enriched was transporter activity (GO: 0005215, p value 0.00046). The overlap between these genes and the transcriptome data was minimal, which we attributed to two potential reasons: first, the presence of *OsNIGT1/4* in the double mutant might inhibit target genes, resulting in less than twofold up regulation; second, the DEGs in the transcriptome might be not direct targets of *OsNIGT2/3* but rather indirect targets. Among the 1,201 potential target genes, thirteen were involved in nitrogen absorption and assimilation (**Table 1**), some of which were also identified via transcriptome analysis (**Figure 5C**). UPM1 is a key enzyme in the biosynthesis of siroheme, a prosthetic group of nitrate reductase (NIR). *Os03g0684700* encodes an integral membrane HPP protein that shares homology with *Arabidopsis* proteins known to be components of the nitrite transport system in plastids (Maeda et al., 2014). *Os11g0264300* and *Os02g0136000* encode NIN-like proteins, which are key regulators of plant responses to nitrogen availability. *OsNIGT1* was present among the potential regulated genes, suggesting an autorepression mechanism among the *OsNIGT* clade proteins. Although *OsNIGT2* and *OsNIGT3* were not identified directly, the B-box motif was found in the promoter regions of *OsNIGT2* (-535 and -748 upstream of ATG) and *OsNIGT3* (-301, -998, -1019, and -1227 upstream of ATG), implying potential autorepression by OsNIGT2/3. Further through Y1H, we found that OsNIGT2/3 can bind to the B-box motif on the promoter of *OsNIGT2/3* (**Supplementary Figure S4**). Additionally, twelve identified genes were involved in phosphorus (Pi) utilization and signal transduction (**Table 1**), including five phosphate transporters (*OsPht1;10*, *OsPht1;9*, *OsPht1;13*, *OsPht1;4*, and *OsMPT*), two SPX proteins (*OsSPX4* and *OsSPX2*), and five phosphate starvation-induced genes (*OsPHR1*, *OsPI1*, etc.). Three other genes (*Os07g0181000*, *Os08g0434300*, and *Os05g0194900*) encode enzymes involved in carbon metabolism, which is also crucial for nitrogen metabolism.

**Table 1:** Potential target genes of *OsNIGT2* regulation identified by DAP-seq analysis.

RAP-ID	-LOG_10_(p value)	Gene symbol	Best-hit-arabi^1^	arabi-symbol	Putative function
Os06g0325200	114.5	OsPht1; 10	AT1G76430.1	PHT1;9	phosphate transporter 1;9
Os03g0827500	46.6	OsSPX4	AT5G15330.1	ATSPX4	SPX domain gene 4
Os02g0620500	38.7	OsAMT1; 3	AT4G13510.1	AMT1;1	ammonium transporter 1;1
Os07g0181000	33.2	OsPK2	AT3G22960.1	PKP1	pyruvate kinase family protein
Os01g0720400	33.0		AT1G73010.1	ATPS2	phosphate starvation-induced gene 2
Os02g0325600	29.1	OsNIGT1	AT1G25550.1	NIGT1.1	myb-like transcription factor family protein
Os11g0264300	25.5	OsNLP5	AT2G43500.1	NLP8	plant regulator RWP-RK family protein
Os02g0620600	28.3	OsAMT1; 2	AT4G13510.1	AMT1;1	ammonium transporter 1;1
Os08g0155400	28.2	OsNRT1.1A	AT1G12110.1	ATNRT1	nitrate transporter 1.1
Os03g0329900	27.0	OsPHR1	AT4G28610.1	AtPHR1	phosphate starvation response 1
Os02g0202200	22.7	OsSPX2	AT5G20150.1	ATSPX1	SPX domain gene 1
Os01g0631200	23.1		AT5G40850.1	UPM1	urophorphyrin methylase 1
Os01g0682001	20.9	OsGOGAT1	AT5G53460.1	GLT1	NADH-dependent glutamate synthase 1
Os06g0324800	21.5	OsPht1; 9	AT1G76430.1	PHT1;9	phosphate transporter 1;9
Os02g0112600	23.0	OsNRT2.2	AT1G08090.1	ATNRT2.1	nitrate transporter 2:1
Os05g0468700	21.3	OsAMT2; 1	AT2G38290.1	AMT2;1	ammonium transporter 2
Os02g0743400	18.1	OsPIN1	AT1G73590.1	ATPIN1	auxin efflux carrier family protein
Os02g0136000	17.6	OsNLP6	AT1G64530.1	NLP6	plant regulator RWP-RK family protein
Os08g0434300	16.7		AT3G47520.1	MDH	malate dehydrogenase
Os08g0468100	13.7	OsNIA1	AT1G77760.1	NIA1	nitrate reductase 1
Os04g0555300	14.5		AT3G47420.1	ATPS3	phosphate starvation-induced gene 3
Os04g0186800	15.2	OsPht1; 13	AT2G32830.1	PHT1;5	phosphate transporter 1;5
Os02g0735200	13.8	OsGS1	AT5G37600.1	ATGLN1;1	glutamine synthase clone R1
Os04g0186400	14.7	OsPht1; 4	AT2G32830.1	PHT1;5	phosphate transporter 1;5
Os05g0194900	14.9		AT4G26270.1	PFK3	phosphofructokinase 3
Os08g0156600	12.1		AT3G47420.1	ATPS3	phosphate starvation-induced gene 3
Os02g0767500	11.2	OsMPT	AT5G14040.1	PHT3;1	phosphate transporter 3;1
Os10g0554200	16.8	OsNRT1.1B	AT1G12110.1	ATNRT1	nitrate transporter 1.1
Os01g0902800	9.3		AT1G32450.1	NRT1.5	nitrate transporter 1.5
Os03g0684700	7.8		AT5G62720.1	AtNITR2;1	integral membrane HPP family protein
Os01g0838350	7.4	OsPI1			
Os03g0749000	8.0		AT2G38290.1	AMT2;1	ammonium transporter 2

^1^ Best-hit-arabi indicates the gene in *Arabidopsis*<.

These genes are involved mainly in nitrogen and phosphorus absorption, utilization and signal transduction, and the rank is based on the p value.

### OsNIGT2 and OsNIGT3 can directly bind the promoters of *NRT* and *AMT*

To investigate whether the B-box sequence mediates the binding of OsNIGT2, a yeast one-hybrid (Y1H) assay was conducted using 400 bp promoter sequences surrounding the peaks of eight potential targets: *NRTs*, *AMTs*, *OsSPX4*, and *OsNIGT1* (**Figure 6C**). The wild-type promoter (wt-pro) represents the native B-box motif-containing sequence (GAATC/AT), whereas the mutant promoter (mu-pro) contains mutated B-box motifs (tAcgC/AT). Both wt-pro and mu-pro were prepared as reporter strains and passed the self-activation test. As shown in **Figure 6C**, clones harboring OsNIGT2 with the wt-pro reporter strain thrived on SD/-Leu/AbA250 medium, whereas the negative control (empty pGADT7 AD) did not grow. Furthermore, no clones with the mu-pro reporter strain grew, indicating that the B-box motif is essential for DNA binding. Additionally, we examined the interaction between these eight promoters and OsNIGT3, revealing that OsNIGT3 also binds to the B-box motif (**Figure 6C**).

**Figure 7:**
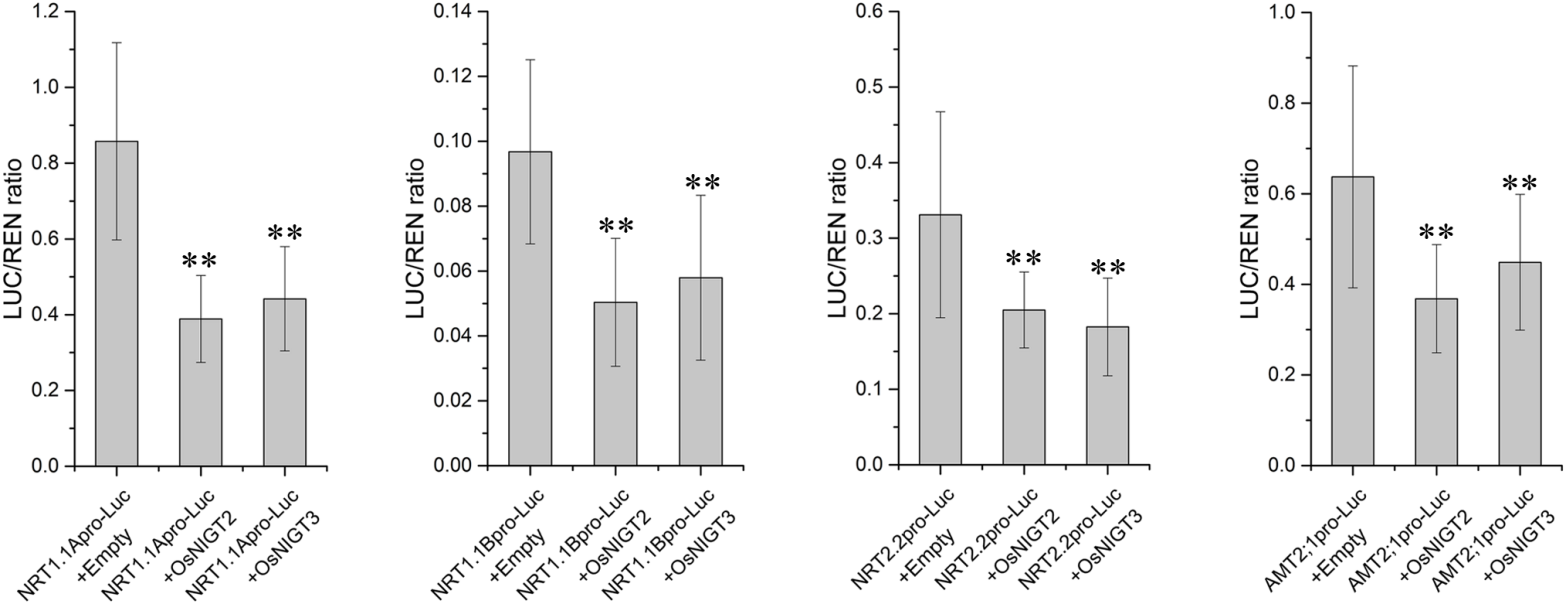
Dual luciferase reporter assay of the interaction of OsNIGT2 and OsNIGT3 with potential promoters. Asterisks indicate significant differences of LUC/REN ratio between the Empty and OsNIGT2/NIGT3 according to two-tailed Student’s t tests: **, P< 0.01.

The transcriptional regulation activity was further validated using a transient luciferase assay (**Figure 7**). Compared with the empty vector, co-expression of *OsNIGT2* and *OsNIGT3* with the promoters of three NRTs and one AMT in tobacco leaves resulted in reduced luciferase (LUC) activity, suggesting that OsNIGT2 and OsNIGT3 repressed the luciferase expression. These data indicate that OsNIGT2 and OsNIGT3 directly regulate the transcription of nitrogen absorption genes.

### *OsNIGT2* and *OsNIGT3* knockout promotes both grain yield and NUE

**Figure 8:**
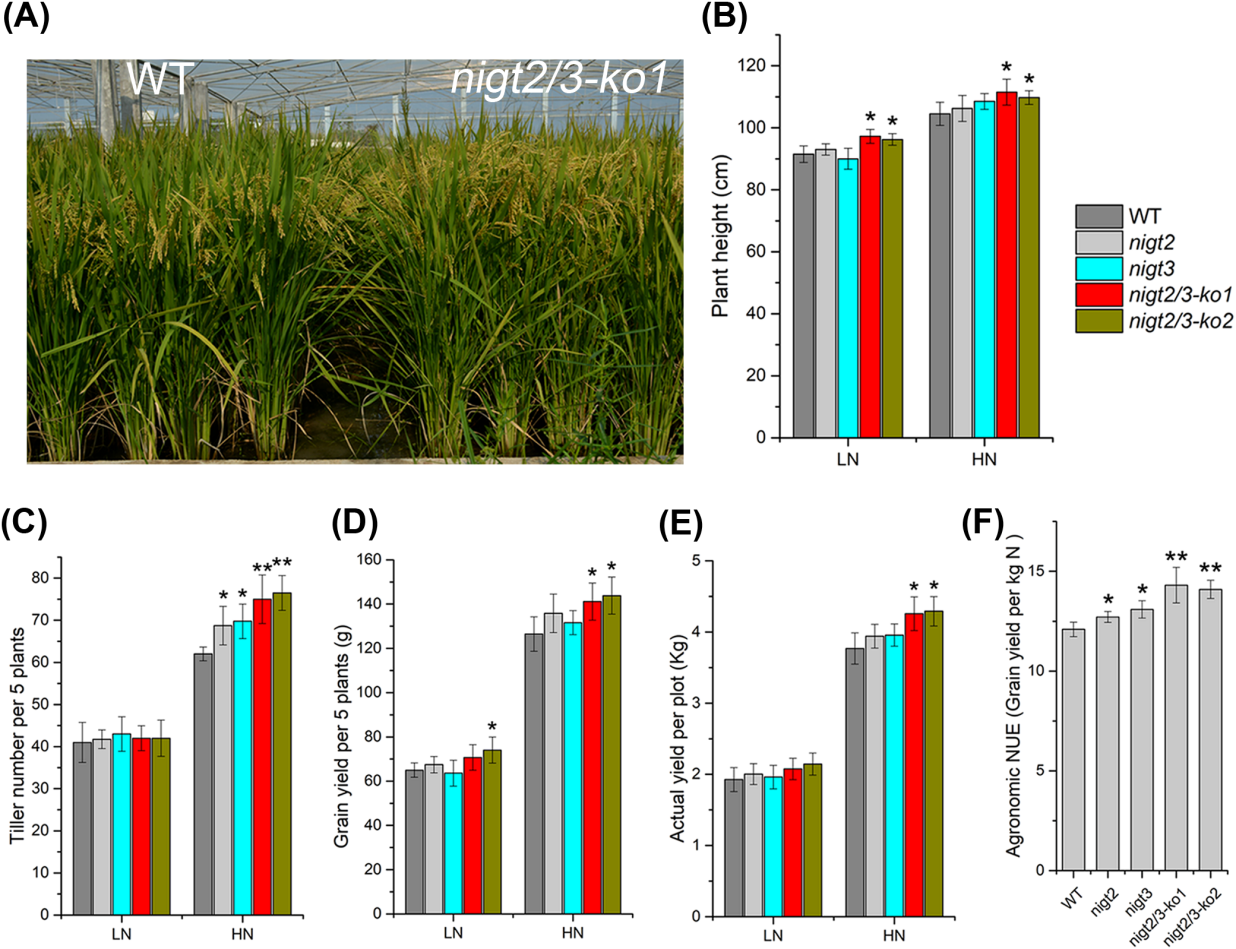
*OsNIGT2* and *OsNIGT3* knockout promotes grain yield and NUE. **(A)** Growth phenotype of wild-type (WT) and *nigt2/3-ko1* plants at maturing stage in the HN field; **(B–E)** Plant height **(B)**, Tiller number per 5 plants **(C)**, Grain yield per 5 plants **(D)**, Actual yield **(E)** of WT and mutants under LN and HN conditions; **(F)** Agronomic NUE of WT and mutants. Values are the means ± SD (four replications), asterisks indicate significant differences between the WT and single *nigt2*, *nigt3*, and dual *nigt2/3* mutants by two-tailed Student’s t-test: *, P < 0.05; **, P < 0.01.

To investigate the role of *OsNIGT2* and *OsNIGT3* in rice NUE, we performed field trials for WT and mutants. As expected, the dual *nigt2/3* mutants displayed improved growth in field trials, the plant height of the dual *nigt2/3* mutants was higher than that of WT (**Figures 8A, B**). The knockout mutants exhibited a significant increase of tiller number under HN compared with the WT, while in LN there was no significant difference (**Figure 8C**). Grain yield per plant and per plot were significantly affected in the dual *nigt2/3* mutants under HN (**Figures 8D, E**). While in other cases, there was no statistically significant difference between mutants and WT, although the values of mutants were slightly higher than that of WT. In addition, compare to WT, agronomic NUE was significantly higher for both single and dual gene mutations (**Figure 8F**). In summary, these results suggest that *OsNIGT2* and *OsNIGT3* negatively regulate NUE and yield in rice.

## Discussion

NUE is a complex agronomic trait that describes the efficiency of nitrogen uptake and utilization by crops. It comprises two key components: N uptake efficiency (NUpE), which is the efficiency of absorption/uptake of supplied nitrogen, and N utilization efficiency (NUtE), which is the efficiency of assimilation and remobilization of plant nitrogen to ultimately produce grain. On the basis of phenotyping and mapping quantitative trait loci (QTLs), allelic variations of some genes in cereal crops, especially rice, have been shown to affect NUE (Liu et al., 2022). Indeed, most of these genes are directly involved in nitrogen uptake, primary assimilation, and remobilization. Additionally, NUE in crops can also be increased by the ectopic overexpression of genes directly involved in nitrogen uptake (Fan et al., 2016; Luo et al., 2023; Wang et al., 2018). TFs, which can coordinately regulate the expression of genes encoding nitrogen transporters and assimilating enzymes, might be particularly attractive targets for improving NUE as they may be able to enhance multiple steps cooperatively. To date, several TFs capable of regulating nitrogen absorption and assimilation, such as *OsNLP1* (Alfatih et al., 2020), *OsNLP4* (Yu et al., 2021), *OsNhd1* (Li et al., 2022), *OsNAC42* (Tang et al., 2019), and *OsLBD37/38/39* (Zhu et al., 2022), have been identified in rice. In the present study, we demonstrated that *OsNIGT2/3* also act as crucial regulators to simultaneously coordinate many processes in nitrogen utilization and signaling pathways.

As major components of HATS, members of the OsNRT2 family respond to nitrogen availability. *OsNIGT2* and *OsNIGT3* act as transcriptional suppressors of *OsNRT2s* under HN conditions (**Figure 5C**). This finding is consistent with the findings that *Arabidopsis HRS1* and *HHO1* negatively control nitrate HATS (Safi et al., 2021). *OsNRT1.1A* and *OsNRT1.1B*, which are components of the nitrate dual-affinity transport system and signal transduction, are responsible for sensing intracellular and environmental nitrogen status, respectively (Wang et al., 2020a). Here, we found that *OsNIGT2* and *OsNIGT3* can inhibit the expression of *OsNRT1.1A* and *OsNRT1.1B* under both LN and HN conditions (**Figure 5C**; **Supplementary Figure S3**). *OsAMT1s* (*OsAMT1.1*-*OsAMT1.3*) belong to the NH^4+^ HATS family, whereas members of the other three subfamilies are characterized as low-affinity transporters. The expression of these genes fluctuates with nitrogen availability, and *OsNIGT2* and *OsNIGT3* can suppress *OsAMT1s* under HN conditions (**Figure 5C**). In addition to ammonium and nitrate transporters, several key genes involved in N assimilation were also upregulated in the double mutant under HN conditions (**Figure 5C**). Five genes (*OsCAT1*, *OsATL7*, *OsLHT7*, *OsAAP6* and *OsAAP7A*; **Supplementary Data S1**) encoding amino acid transporters were also upregulated in the double mutant under HN conditions. *OsAAP6* functions as an important regulator of root amino acids and ultimately with the biosynthesis and deposition of grain proteins (Wang et al., 2020c). The upregulated of these five genes might be due to the enhanced absorption and assimilation of N.

Nitrogen (N) and phosphorus (Pi) are the two most abundant mineral nutrients utilized by plants, and their coordinated utilization is essential for maintaining optimal plant growth and achieving maximal crop yields. Since *AtNIGT1/HRS1* was first found to integrate N and Pi signals in roots (Medici et al., 2015), seven studies have analyzed the molecular mechanism of the NIGT1 clade in N and Pi metabolism (Ueda et al., 2020a, 2020b). Pi starvation increases the accumulation of *AtNIGT1/HRS1*, and AtNIGT1 can coordinate N–Pi acquisition under fluctuating nutritional conditions. Specifically, AtNIGT1 can target repressor genes of the nitrate response and phosphate starvation response (*PHT1;2/1;4*, *SPX1/2/4*, and *PHO2*), thus coordinating N and Pi utilization (Ueda et al., 2020a; Wang et al., 2020b). Pi starvation induces *OsNIGT1*, and *OsNIGT1* represses plant growth and mitigates Pi starvation signaling by binding directly to the promoters of marker genes, such as *IPS1* and *SPX2* (Zhang et al., 2023). Here, we found that *OsNIGT2* can regulate genes involved in Pi uptake and signal transduction, such as Pi transporters, *OsSPXs*, and *OsPHR1* (**Table 1**). These findings suggest that *OsNIGT2/3* can also coordinate the utilization of N and Pi. In this study, we did not explore the growth of the mutant under different Pi concentrations, and the effect of *OsNIGT2/3* under low-Pi conditions remains to be further studied.

The nitrogen concentration in the soil is uneven and is prone to be flow. When the external N concentration is lower than the demand of plant, the root system needs to maintain a relatively high level of expression of genes related to N transport. Conversely, when the external N concentration is higher than the demand of plant, the root system needs to maintain a lower level of expression of genes related to N transport. At this time, the *OsNIGT2* and *OsNIGT3* exert their feedback inhibition function, that is to say *OsNIGTs* play a braking role in the process of plants’ response to nitrogen. Through hydroponic experiments, we found that the dry weight, nitrogen (N) content, total amino acid content, and nitrate content of the *nigt2/3* double mutants were higher than those of the wild type, whereas the single mutants presented no significant differences (**Figure 3**). Compared with the wild type, the *hrs1;hho1;hho2;hho3* quadruple mutant has larger shoots, and the *hrs1;hho1* double mutant also has larger shoots, although its phenotype is less pronounced than that of the quadruple mutant (Safi et al., 2021). Our physiological observations and the phenotypes observed in *Arabidopsis* suggest that loss-of-function mutations in NIGT can increase N uptake and biomass, especially under conditions of sufficient nitrogen. Notably, *OsNIGT2* and *OsNIGT3* maintain certain expression levels under LN conditions (**Figures 1A**, **5C**); however, the reason that they do not inhibit the expression of their targets under LN conditions remains to be elucidated.

Through field tests, we found that the dual *nigt2/3* mutant exhibit improved grain yield, while there was no difference in single gene mutations compared to the WT under HN (**Figure 8**). The dual *nigt2/3* mutants increase the yield by increasing the plant height and tiller number, which may be due to the increased N absorption. Under LN condition, most mutants (single and dual) and WT do not show significant differences in yield. With the exception of one dual mutant, the grain yield per 5 plants was higher than that of WT, but no significant difference in plot yield between them (**Figure 8**). Elimination of *OsHHO3* (*OsNIGT3*) improves growth potential and yield under N-deficient, but no difference under regular condition (Liu et al., 2023), which seems to contradict the results of our research. The possible reasons are different planting environment and nitrogen application level. Liu et al. grow rice in greenhouse, while we choose field. Our HN condition may be similar to the regular condition of Liu et al., and we agree with Liu et al. that single gene mutations do not significantly increase yield under HN condition. Actually, we found that the production of dual mutant was significantly increased, indicating that there may be functional redundancy between the two genes. Our LN condition was extremely low (no nitrogen fertilizer), which may lower than the N-deficient condition of Liu et al. The LN condition greatly limits the growth of rice, which may resulting in little difference in N uptake between the mutations and WT. The agronomic NUE of the mutants (single and dual) were higher than that of WT (**Figure 8F**), indicating that *OsNIGT2* and *OsNIGT3* knockout improve N utilization efficiency. Based on this result, its need to detect the variants of *OsNIGT2* and *OsNIGT3* in the backbone cultivars, and test whether *OsNIGT2* and *OsNIGT3* knockout can improve the NUE and yield of cultivars, for further proof of their utilization value in rice breeding.

In conclusion, our results demonstrate that *OsNIGT2* and *OsNIGT3* acts as a pivotal regulator of NUE by coordinating N uptake, assimilation and signaling, and is a promising candidate gene for improving crop yield and NUE.

## Data Availability

The data presented in the study are deposited in the Genome Sequence Archive (GSA, https://ngdc.cncb.ac.cn/gsa/) of the China National Center for Bioinformation (CNCB), accession number: CRA026752 and CRA026749.
