# Supplementary material for: Rice transcription factors OsNIGT2/3 regulate nitrogen acquisition by repressing OsNRTs and OsAMTs under high-nitrogen conditions
Source: Front Plant Sci. 2025 Jul 2;16:1591808. doi: 10.3389/fpls.2025.1591808 (PMC12263960; doi:10.3389/fpls.2025.1591808)
Supplement: Supplementary file 1 [file DataSheet1.docx]

**Supplemental Method**

**Plant materials preparation and DEGs analysis of RNA-Seq data**

The *japonica* variety Nipponbare were germinated on filter paper soaked in water for 5 days with a 16/8 h light/dark photoperiod, and then the uniform seedlings were transferred into coconut matrix. The basal solution consisted of the following nutrient compounds: K_2_SO_4_ (0.35 mM), KH_2_PO_4_ (0.3 mM), CaCl_2_·2H_2_O (1 mM), MgSO_4_·7H_2_O (1 mM), Na_2_SiO_3_·9H_2_O (0.5 mM), H_3_BO_3_ (20 μM), MnCl_2_·4H_2_O (9 μM), ZnSO_4_·7H_2_O (0.76 μM), CuSO_4_·5H_2_O (0.32 μM), (NH_4_)_6_Mo_7_O_24_·4H_2_O (0.06 μM) and Fe-EDTA (40 μM), with the pH adjusted to 5.5. For the normal growth before treatment, 0.5 mM NH_4_NO_3_ was added to the basal nutrient solution. Fresh medium was renewed every two days to maintain a steady nutritional state and optimal pH levels, and the seedlings were grown to the five-leaf stage. Then plants were transferred to fresh basal nutrient solution (N free) for one week. Plants were transiently treated at 9:00 (2 h after the start of light cycle) by adding N at a final concentration of 2.5 mM NH_4_NO_3_. The roots were harvested at 0 (pre-treatment), 0.5, 1, 2, 4, 12, and 24 h after treatments.

Differentially expressed genes (DEGs) were identified using DEseq2 based on a comparison of the treatment time points (0.5 and 1h) and pre-treatment (0 h) with adjusted p-value < 0.01, meanwhile absolute log_2_ of fold change of between treatment and control value > 1. DEGs associated with early ammonium nitrate-responsive (0.5 and 1h) were used to analyze similar expression patterns during the N starvation and the ammonium nitrate treatments. This analysis yielded a set of 825 and 1850 DEGs in Nipponbare at 0.5 and 1 h, respectively (Supplemental Data S3).

**Supplemental Figures and Tables**


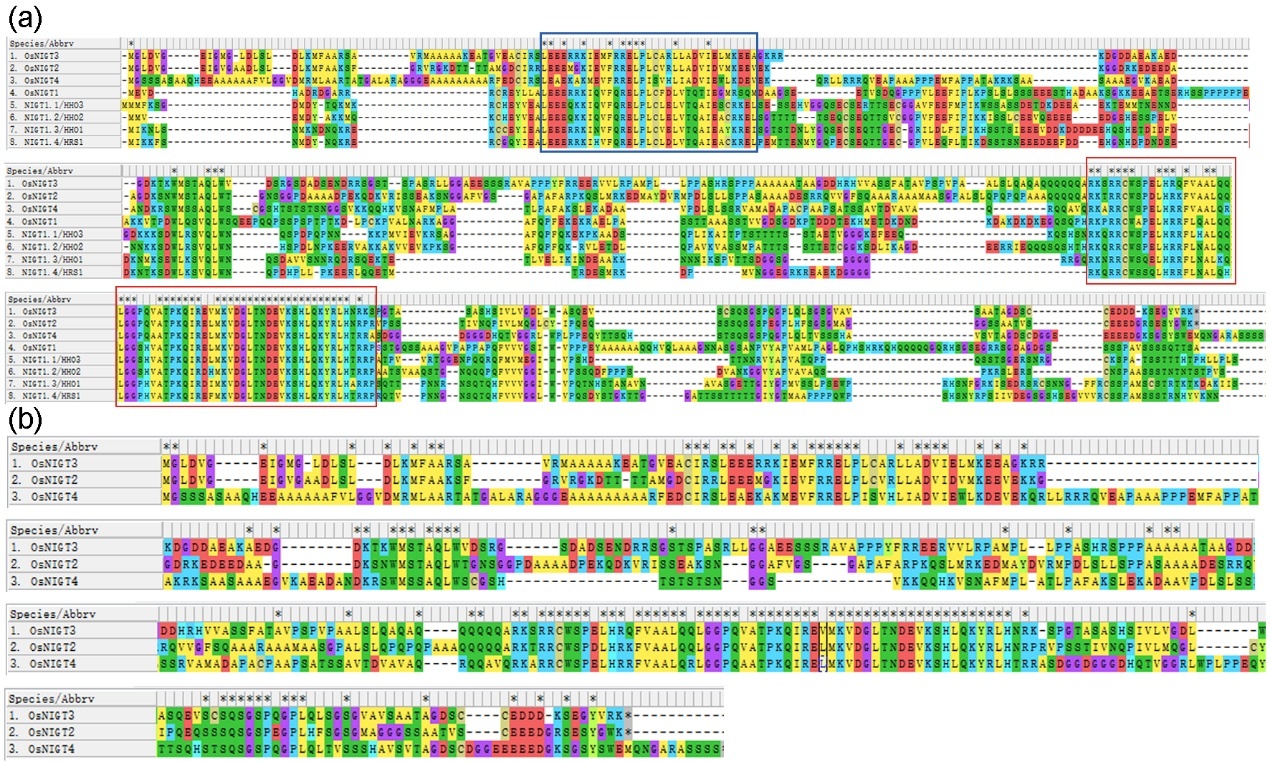


**Figure S1** **Comparison** **protein sequence of *OsNIGT2*/3/4 with other NIGTs**

(a) protein sequences of *OsNIGT2/3/4* with other NIGTs, the blue box represents the HGD motif (hydrophobic and globular domain), the red box represents the GARP motif (B-motif); (b) protein sequences of *OsNIGT2/3/4*.


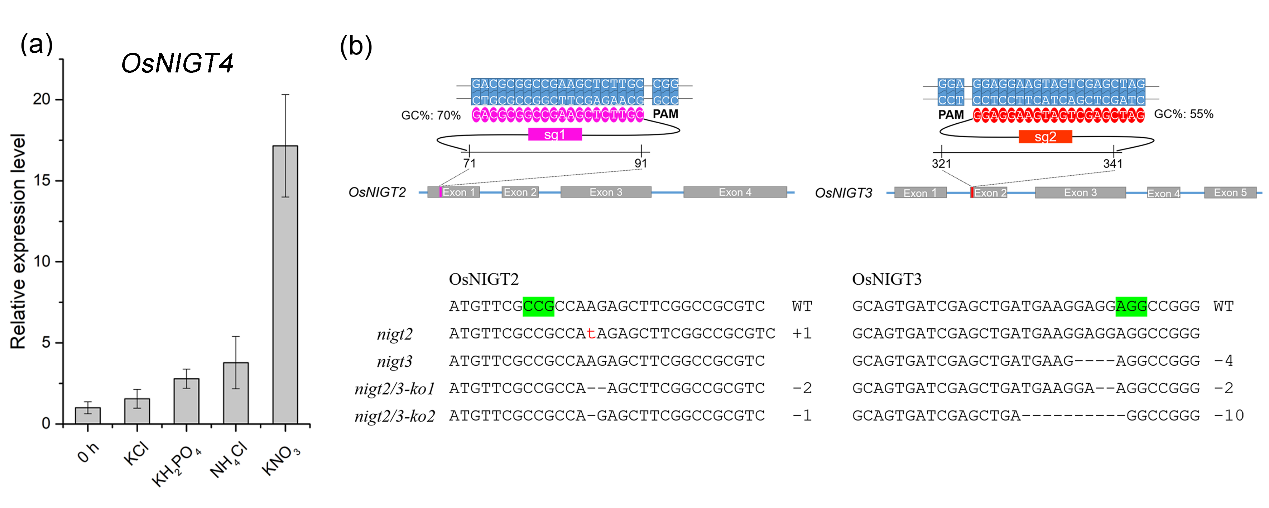


**Figure S2 Nitrate-specific induction of *OsNIGT4* expression and Mutant of *OsNIGT2*/3 obtained by CRISPR/Cas9**

(a) Nitrate-specific induction of OsNIGT4 expression; (b) CRISPR/Cas9 target site and mutant sequences of *nigt2* and *nigt3* target sites.


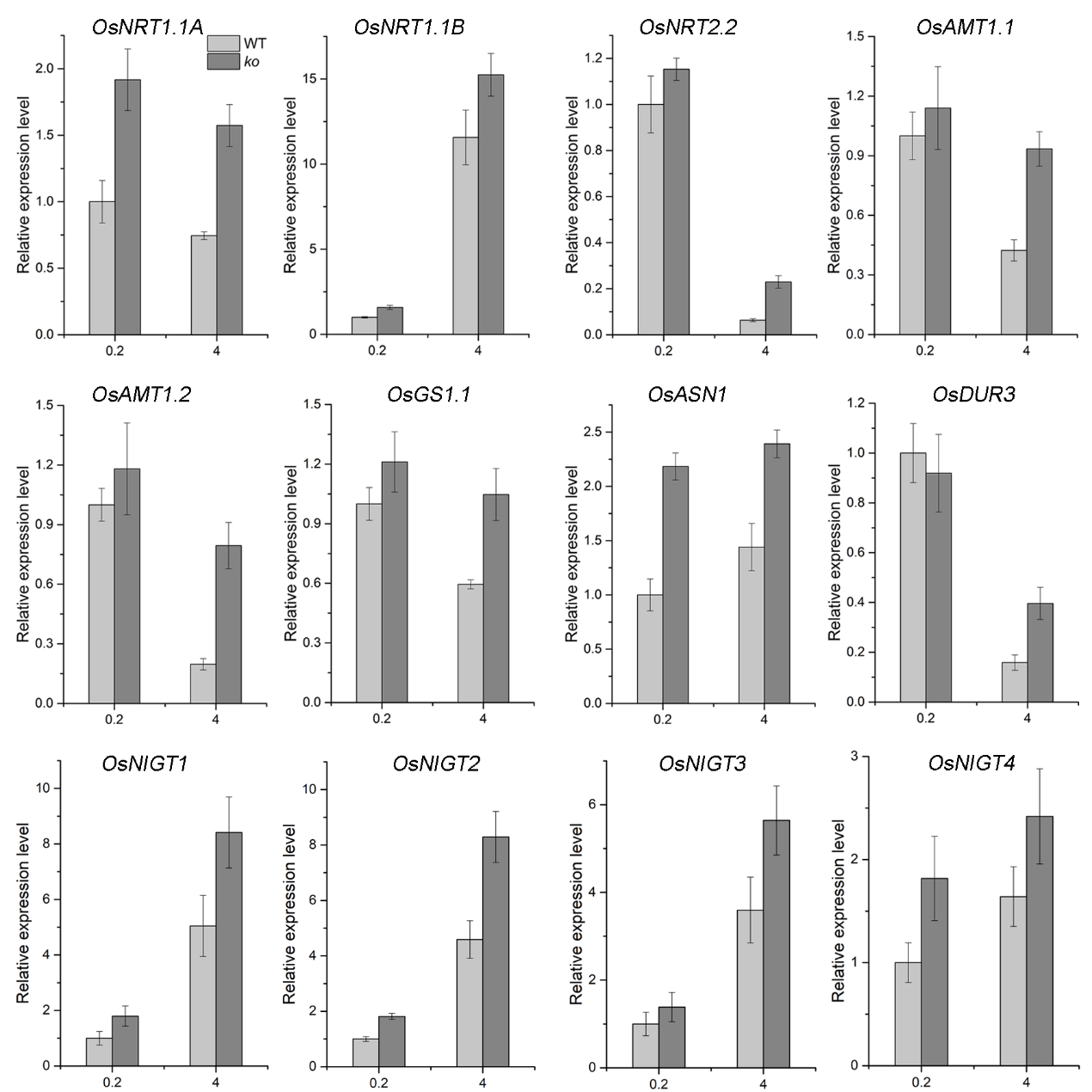


**Figure S3 RT-qPCR validation of transcriptome data**


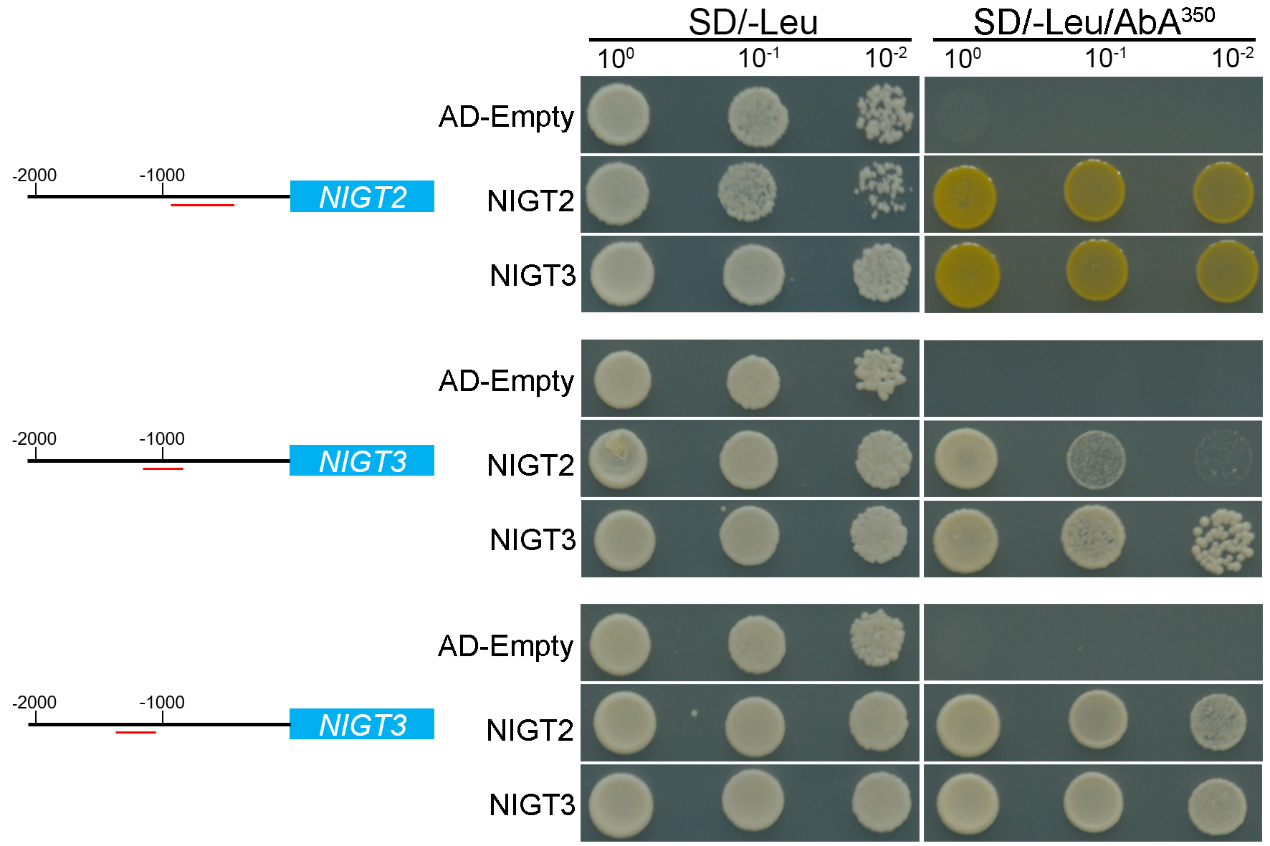


**Figure S4 Y1H assay of OsNIGT2 and OsNIGT3 interactions with the potential b-box of *OsNIGT2/3* promoters.**

**Supplemental Table S1 79 differentially regulated transcription factors in the transcriptome of rice roots following NH₄NO₃ resupply after nitrogen deficiency**

| log_2_FC | p value | Locus ID | Best-hit-arabi | arabi-symbol | Family |
| --- | --- | --- | --- | --- | --- |
| -5.15461 | 1.49E-246 | LOC_Os03g33090 | AT5G67420.1 | ASL39 | LBD |
| -4.58036 | 4.17E-107 | LOC_Os11g08210 | AT5G08790.1 | anac081 | NAC |
| -8.3896 | 4.20E-99 | LOC_Os05g02390 | AT5G59820.1 | RHL41 | C2H2 |
| -4.10602 | 1.09E-92 | LOC_Os07g02800 | AT2G03500.1 |  | G2-like |
| -4.6096 | 3.36E-73 | LOC_Os02g22020 | AT1G25550.1 |  | G2-like |
| -5.58926 | 2.39E-69 | LOC_Os03g41330 | AT5G67420.1 | ASL39 | LBD |
| -4.46253 | 3.02E-67 | LOC_Os01g43550 | AT4G01250.1 | AtWRKY22 | WRKY |
| -1.97315 | 7.56E-33 | LOC_Os07g40000 | AT5G67420.1 | ASL39 | LBD |
| -2.78158 | 4.10E-31 | LOC_Os04g56990 | AT5G06800.1 |  | G2-like |
| -3.34264 | 1.75E-25 | LOC_Os03g55590 | AT1G13300.1 | HRS1 | G2-like |
| -1.89193 | 2.54E-23 | LOC_Os05g37730 | AT2G38090.1 |  | MYB |
| -2.12344 | 1.02E-22 | LOC_Os04g42950 | AT4G13480.1 | AtMYB79 | MYB |
| -3.79794 | 1.31E-21 | LOC_Os01g54890 | AT5G47220.1 | ATERF-2 | ERF |
| -2.72315 | 4.49E-19 | LOC_Os03g62230 | AT1G34370.1 | STOP1 | C2H2 |
| -3.80713 | 8.38E-19 | LOC_Os02g49560 | AT3G30530.1 | ATBZIP42 | bZIP |
| -2.9018 | 2.29E-18 | LOC_Os05g45020 | AT2G25900.1 | ATCTH | C3H |
| -1.64164 | 1.17E-17 | LOC_Os05g38140 | AT5G54680.1 | bHLH105 | bHLH |
| -1.38631 | 1.35E-17 | LOC_Os02g52780 | AT3G19290.1 | ABF4 | bZIP |
| -1.01053 | 3.31E-17 | LOC_Os10g41460 | AT2G44730.1 |  | Trihelix |
| 1.106597 | 7.79E-17 | LOC_Os05g51160 | AT5G47390.1 |  | MYB_related |
| -1.66481 | 2.34E-16 | LOC_Os12g39640 | AT2G03500.1 |  | G2-like |
| -1.14109 | 3.15E-11 | LOC_Os01g04930 | AT2G38090.1 |  | MYB |
| -1.59697 | 3.89E-11 | LOC_Os04g49230 | AT2G46870.1 | NGA1 | B3 |
| -2.07721 | 4.73E-11 | LOC_Os03g20910 | AT3G03660.1 | WOX11 | WOX |
| -3.31274 | 2.48E-10 | LOC_Os01g60020 | AT5G63790.1 | ANAC102 | NAC |
| -1.8362 | 1.26E-08 | LOC_Os07g12340 | AT5G63790.1 | ANAC102 | NAC |
| -2.22358 | 3.52E-08 | LOC_Os01g61080 | AT5G07100.1 | WRKY26 | WRKY |
| 1.446809 | 9.73E-08 | LOC_Os02g49440 | AT5G60850.1 | OBP4 | Dof |
| 1.993885 | 1.36E-07 | LOC_Os01g03890 | AT3G02550.1 | LBD41 | LBD |
| -1.59678 | 1.48E-07 | LOC_Os02g13800 | AT3G24520.1 | AT-HSFC1 | HSF |
| -3.3987 | 1.91E-07 | LOC_Os02g43330 | AT2G46680.1 | ATHB-7 | HD-ZIP |
| -2.21923 | 2.79E-07 | LOC_Os06g06360 | AT4G23810.1 | ATWRKY53 | WRKY |
| -1.41563 | 2.82E-07 | LOC_Os08g38220 | AT1G51700.1 | ADOF1 | Dof |
| 1.664236 | 5.00E-07 | LOC_Os08g43334 | AT5G62020.1 | AT-HSFB2A | HSF |
| -1.92697 | 5.10E-07 | LOC_Os01g11910 | AT2G40200.1 |  | bHLH |
| -1.50326 | 1.46E-06 | LOC_Os02g46030 | AT5G17300.1 | RVE1 | MYB_related |
| -1.15185 | 2.30E-06 | LOC_Os01g04020 | AT1G68550.1 |  | ERF |
| 1.45943 | 3.88E-06 | LOC_Os10g25170 | AT1G72360.3 | HRE1 | ERF |
| -1.5021 | 7.70E-06 | LOC_Os01g06640 | AT2G40200.1 |  | bHLH |
| -1.11358 | 1.90E-05 | LOC_Os06g14190 | AT1G10170.1 | ATNFXL1 | NF-X1 |
| -1.07587 | 1.90E-05 | LOC_Os12g37970 | AT5G59780.3 | ATMYB59 | MYB |
| -2.76642 | 1.92E-05 | LOC_Os03g17150 | AT2G37430.1 |  | C2H2 |
| -2.51295 | 2.75E-05 | LOC_Os02g40530 | AT4G13480.1 | AtMYB79, | MYB |
| 1.882266 | 2.87E-05 | LOC_Os06g17410 | AT5G60850.1 | OBP4 | Dof |
| -1.33696 | 6.55E-05 | LOC_Os10g23050 | AT1G72210.1 |  | bHLH |
| 2.389929 | 0.000148 | LOC_Os04g43560 | AT4G28530.1 | NAC074 | NAC |
| 1.145217 | 0.000151 | LOC_Os04g02510 | AT1G75710.1 |  | C2H2 |
| 1.589058 | 0.000168 | LOC_Os08g38990 | AT5G56270.1 | WRKY2 | WRKY |
| 1.2442 | 0.000184 | LOC_Os11g47460 | AT5G59780.3 | ATMYB59 | MYB |
| -1.19936 | 0.00019 | LOC_Os02g04340 | AT1G64530.1 |  | Nin-like |
| -1.95006 | 0.000332 | LOC_Os04g34970 | AT5G61890.1 |  | ERF |
| 1.182337 | 0.000345 | LOC_Os09g36910 | AT2G17770.2 | ATBZIP27 | bZIP |
| -2.35129 | 0.000359 | LOC_Os07g39470 | AT1G50600.1 | SCL5 | GRAS |
| -1.67716 | 0.000418 | LOC_Os08g29660 | AT4G23810.1 | ATWRKY53 | WRKY |
| -1.56898 | 0.000472 | LOC_Os07g48870 | AT5G49620.1 | AtMYB78 | MYB |
| 2.359545 | 0.000508 | LOC_Os05g50340 | AT1G75250.2 | ATRL6 | MYB_related |
| 1.110468 | 0.000598 | LOC_Os03g06630 | AT3G22830.1 | AT-HSFA6B | HSF |
| 1.200043 | 0.000683 | LOC_Os03g20900 | AT3G04030.3 |  | G2-like |
| -1.0045 | 0.000746 | LOC_Os04g45810 | AT2G46680.1 | ATHB-7 | HD-ZIP |
| -1.92245 | 0.000835 | LOC_Os01g53220 | AT3G24520.1 | AT-HSFC1 | HSF |
| 1.397472 | 0.000877 | LOC_Os11g06180 | AT4G08250.1 |  | GRAS |
| 1.875184 | 0.000983 | LOC_Os05g40960 | AT5G18240.4 | ATMYR1 | G2-like |
| -1.20724 | 0.001189 | LOC_Os04g49110 | AT5G52510.1 | SCL8 | GRAS |
| 1.032039 | 0.001193 | LOC_Os11g03540 | AT3G54320.1 | ASML1 | AP2 |
| -2.18093 | 0.002566 | LOC_Os01g43590 | AT3G24520.1 | AT-HSFC1 | HSF |
| -1.50645 | 0.002566 | LOC_Os02g53150 | AT1G11510.1 |  | GeBP |
| 1.023075 | 0.002751 | LOC_Os05g48850 | AT4G28500.1 | ANAC073 | NAC |
| 1.030718 | 0.00297 | LOC_Os05g06340 | AT5G26930.1 | GATA23 | GATA |
| -1.50507 | 0.003101 | LOC_Os05g49620 | AT4G11070.1 | AtWRKY41 | WRKY |
| -2.35954 | 0.003247 | LOC_Os05g04210 | AT5G49620.1 | AtMYB78 | MYB |
| -1.69366 | 0.003425 | LOC_Os11g05740 | AT5G06250.2 |  | B3 |
| -1.04262 | 0.003554 | LOC_Os02g16540 | AT5G52830.1 | ATWRKY27 | WRKY |
| -2.67594 | 0.003754 | LOC_Os03g60560 | AT5G59820.1 | RHL41 | C2H2 |
| -1.32084 | 0.004195 | LOC_Os10g22600 | AT1G78080.1 | RAP2.4 | ERF |
| -1.18764 | 0.004433 | LOC_Os02g43170 | AT1G75540.1 | STH2 | DBB |
| 1.023654 | 0.00538 | LOC_Os02g34970 | AT5G64530.1 | ANAC104 | NAC |
| -1.21728 | 0.005502 | LOC_Os08g44830 | AT5G22890.1 |  | C2H2 |
| -1.14123 | 0.006726 | LOC_Os09g33550 | AT2G33500.1 |  | CO-like |
| -1.21576 | 0.009527 | LOC_Os02g53690 | AT3G13960.1 | AtGRF5 | GRF |

**Supplemental Table S2** Primers used for gene cloning and plasmid construction.

| **Gene name** | **Forward primer (5’-3’)** | **Reverse primer (5’-3’)** |
| --- | --- | --- |
| OsNIGT2 | CCATCAATCCGTCCACAATCAA | CGCAATTAACCTAACCCACCAA |
| OsNIGT3 | CTTCCTCCAGTTCTTGTTCTTGT | CAACACTACCATCCTCCTCAGA |
| OsNIGT2pro | GATCTCTGCCTTCGACTCCA | TTGATTGTGGACGGATTGATGG |
| OsNIGT3pro | AGACGCTCTTCTTAGGTGGATG | ACAAGAACAAGAACTGGAGGAAG |
| OsNRT1.1Apro | GTGAATACGCTCTAGCACAAGAT | CAAACGCAGGTGAGAGTGAGA |
| OsNRT1.1Bpro | GTTAGAAGTTTGTGTGCGTAGGA | GCGATGGATGCTGTGATGAAG |
| OsNRT2.2pro | ACTGGACACATATCTGCAACTTG | GCTCTAGCCTCTTGGATTGATTG |
| OsAMT1.2pro | GACTATGACCTAACCTCAATG | TATCCGCGCACGTTGCCATCCT |
| OsAMT1.3pro | TTCAGTCAGACACGAGTTCTTCA | GGGCAGAACACATTGGAATCC |
| OsAMT2.1pro | CTACCTCGCCTAGCACCTCTT | CACGGACGAATCGCTACTTG |
| OsNIGT1pro | CGTGCTAGAGATTCCATACCAA | GAAGGAAGGAAGGAACAAGGAAG |

**Supplemental Table S3** Primers used for quantitative real-time PCR.

| **Gene name** | **Gene ID** | **Forward primer (5’-3’)** | **Reverse primer (5’-3’)** |
| --- | --- | --- | --- |
| OsNIGT1 | LOC_Os02g22020 | CAAGTCAGGCAAGAAGGAGGA | GGTAGGAGTTGGGCTAGGAGA |
| OsNIGT2 | LOC_Os07g02800 | CGCAAGTTCGTCGCCGCCTT | CCCTTGCATGAGCACAATCGGT |
| OsNIGT3 | LOC_Os03g55590 | CCGTGCCGTCACCCGTCCC | CGACCAGCACCGCCTCGACT |
| OsNRT1.1A | LOC_Os08g05910 | CCCACACCAAGCAATTCAGG | GTCTTCACCTCCTCCACGTC |
| OsNRT1.1B | LOC_Os10g40600 | GGCAGGCTCGACTACTTCTA | AGGCGCTTCTCCTTGTAGAC |
| OsNRT2.2 | LOC_Os02g02190 | CGGAGCACGCCTAATTAAGAG | GCCTTTCTCCATGACGACATAC |
| OsAMT1.1 | LOC_Os04g43070 | GTCATCTTCGGGTGGGTCAG | CACTTGGTTGTTGCTGTTGGA |
| OsAMT1.2 | LOC_Os02g40710 | GTCCTTCACCACCATCTCCAA | TCAGCCATCTCTTGCCGTAG |
| OsNIR1 | LOC_Os01g25484 | CTCATCGACGAACTTGTGAACCA | ATTCCCTGCAATGCCAACACC |
| OsNR2 | LOC_Os02g53130 | ATCCTCGCCTACATGCAGAAC | GCGGTAATGGTAGTAGCTCTCC |
| OsGS1;1 | LOC_Os02g50240 | CACCAACAAGAGGCACAATG | ACTCCCACTGTCCTGGCAT |
| OsASN1 | LOC_Os03g18130 | CCATCACGAGTTCCACTTCAC | CGGACAGCACCATCTTAACC |
| loricrin | LOC_Os04g54830 | CAAGCCACAACAATGATCGAG | AGAAGTGGCAGCACATAACCA |
| OsUPM1 | LOC_Os01g44050 | TCTCCAACGACGTGCTCGAT | GCCGCCTTTCAACCGCACCA |
| OsBT | LOC_Os01g68020 | GCCTTCGTCCGCCTCCTCTACGC | CCCCTCGCACCGCCGCTTC |
| OsLBD38 | LOC_Os03g41330 | CGTCTTCGTCGCCAAGTTCT | CAGTTGCCCGTCCACATGAG |
| OsDUR3 | LOC_Os10g42960 | CTCGTCGTCTTCGTCTTCCT | GAGCTGAGCATGGTGAGGTA |
| OsAAP6 | LOC_Os07g04180 | GTGACTGTTGGAGTGGATGTG | CGATGAACGACGCCTTCTTC |
| OsPIN9 | LOC_Os01g58860 | ATCTGGATGGCGGTGAAGAA | CGGTGGTACGAATGGTGAAC |
| eIF4a | AK073620 | TCACCGACAAGATGAGGAGCAG | CGATACCACGAGCAAGCAGATC |

**Supplemental Table S4**

| Dry weights | | | | |  | Total N concentrations | | | | |
| --- | --- | --- | --- | --- | --- | --- | --- | --- | --- | --- |
| Tissue | N level (mM) | material type | Weight (g) | P value |  | Tissue | N level (mM) | material type | N con. (mg/g DW) |  |
| Roots | 0.2 | WT | 0.0646±0.0059 |  |  | Roots | 0.2 | WT | 11.937±0.216 |  |
|  |  | *nigt2* | 0.0711±0.0032 | 0.0257* |  |  |  | *nigt2* | 12.396±0.362 | 0.1324 |
|  |  | *nigt3* | 0.0699±0.0079 | 0.0998 |  |  |  | *nigt3* | 12.190±0.277 | 0.2810 |
|  |  | *nigt2/3-1* | 0.0725±0.0067 | 0.0376* |  |  |  | *nigt2/3-1* | 12.924±0.224 | 0.0053** |
|  |  | *nigt2/3-2* | 0.0732±0.0062 | 0.0221* |  |  |  | *nigt2/3-2* | 12.830±0.280 | 0.0120* |
|  | 2 | WT | 0.0874±0.0090 |  |  |  | 2 | WT | 21.924±1.036 |  |
|  |  | *nigt2* | 0.0937±0.0079 | 0.1899 |  |  |  | *nigt2* | 23.115±0.499 | 0.0430* |
|  |  | *nigt3* | 0.0925±0.0085 | 0.2964 |  |  |  | *nigt3* | 22.973±0.478 | 0.0668 |
|  |  | *nigt2/3-1* | 0.0998±0.0087 | 0.0219* |  |  |  | *nigt2/3-1* | 25.290±0.930 | 0.0002** |
|  |  | *nigt2/3-2* | 0.0993±0.0113 | 0.0500* |  |  |  | *nigt2/3-2* | 25.800±1.099 | 0.0002** |
|  | 4 | WT | 0.0963±0.0146 |  |  |  | 4 | WT | 28.070±0.612 |  |
|  |  | *nigt2* | 0.1020±0.0092 | 0.6380 |  |  |  | *nigt2* | 29.029±0.328 | 0.0115* |
|  |  | *nigt3* | 0.0999±0.0072 | 0.7028 |  |  |  | *nigt3* | 28.818±0.644 | 0.0895 |
|  |  | *nigt2/3-1* | 0.1096±0.0121 | 0.0319* |  |  |  | *nigt2/3-1* | 31.337±0.906 | 5.5E-05** |
|  |  | *nigt2/3-2* | 0.1099±0.0065 | 0.0122* |  |  |  | *nigt2/3-2* | 30.727±0.600 | 4E-05** |
| Shoots | 0.2 | WT | 0.1705±0.0065 |  |  | Shoots | 0.2 | WT | 15.289±0.403 |  |
|  |  | *nigt2* | 0.1788±0.0054 | 0.0234* |  |  |  | *nigt2* | 15.396±0.478 | 0.7271 |
|  |  | *nigt3* | 0.1822±0.0125 | 0.0470* |  |  |  | *nigt3* | 15.653±0.204 | 0.1098 |
|  |  | *nigt2/3-1* | 0.1898±0.0072 | 0.0002** |  |  |  | *nigt2/3-1* | 16.359±0.350 | 0.0042** |
|  |  | *nigt2/3-2* | 0.1950±0.0127 | 0.0007** |  |  |  | *nigt2/3-2* | 16.055±0.342 | 0.0156* |
|  | 2 | WT | 0.3556±0.0516 |  |  |  | 2 | WT | 34.382±1.209 |  |
|  |  | *nigt2* | 0.3712±0.4595 | 0.5611 |  |  |  | *nigt2* | 35.627±1.492 | 0.2424 |
|  |  | *nigt3* | 0.3841±0.0373 | 0.2594 |  |  |  | *nigt3* | 35.046±1.849 | 0.5696 |
|  |  | *nigt2/3-1* | 0.4099±0.0274 | 0.0303* |  |  |  | *nigt2/3-1* | 37.212±0.386 | 0.0043** |
|  |  | *nigt2/3-2* | 0.4270±0.0475 | 0.0195* |  |  |  | *nigt2/3-2* | 37.084±0.480 | 0.0060** |
|  | 4 | WT | 0.4182±0.0486 |  |  |  | 4 | WT | 37.635±0.710 |  |
|  |  | *nigt2* | 0.4514±0.0487 | 0.2266 |  |  |  | *nigt2* | 38.850±1.160 | 0.1248 |
|  |  | *nigt3* | 0.4478±0.0608 | 0.3342 |  |  |  | *nigt3* | 38.774±1.126 | 0.1381 |
|  |  | *nigt2/3-1* | 0.4831±0.0432 | 0.0216* |  |  |  | *nigt2/3-1* | 40.526±0.903 | 0.0024** |
|  |  | *nigt2/3-2* | 0.4908±0.0623 | 0.0318* |  |  |  | *nigt2/3-2* | 40.795±0.517 | 0.0004** |

Continued

| Total amino acid concentrations | | | | |  | NO_3_^¯^ concentrations | | | | |
| --- | --- | --- | --- | --- | --- | --- | --- | --- | --- | --- |
| Tissue | N level (mM) | material type | con. (mg/g FW) | P value |  | Tissue | N level (mM) | material type | con. (μg/g FW) | P value |
| Roots | 0.2 | WT | 0.1779±0.0216 |  |  | Roots | 0.2 | WT | 38.227±3.439 |  |
|  |  | *nigt2* | 0.2567±0.0218 | 9.64E-09** |  |  |  | *nigt2* | 41.173±4.322 | 0.0782 |
|  |  | *nigt3* | 0.2536±0.0359 | 2.72E-06** |  |  |  | *nigt3* | 42.920±4.898 | 0.0126* |
|  |  | *nigt2/3-1* | 0.2630±0.0289 | 4.26E-08** |  |  |  | *nigt2/3-1* | 46.587±5.275 | 1.40E-04** |
|  |  | *nigt2/3-2* | 0.2768±0.0388 | 1.09E-07** |  |  |  | *nigt2/3-2* | 46.858±6.700 | 4.30E-04** |
|  | 2 | WT | 1.0027±0.0942 |  |  |  | 2 | WT | 587.71±60.15 |  |
|  |  | *nigt2* | 1.0857±0.0774 | 0.0319* |  |  |  | *nigt2* | 627.63±67.66 | 0.1505 |
|  |  | *nigt3* | 1.1207±0.1408 | 0.0246* |  |  |  | *nigt3* | 624.10±46.68 | 0.1178 |
|  |  | *nigt2/3-1* | 1.1596±0.1295 | 0.0016** |  |  |  | *nigt2/3-1* | 655.62±64.09 | 0.0138* |
|  |  | *nigt2/3-2* | 1.2044±1.1694 | 0.0026** |  |  |  | *nigt2/3-2* | 664.13±52.43 | 0.0031** |
|  | 4 | WT | 1.0746±0.1444 |  |  |  | 4 | WT | 745.32±80.81 |  |
|  |  | *nigt2* | 1.2111±0.0774 | 0.0244* |  |  |  | *nigt2* | 807.03±72.34 | 0.0766 |
|  |  | *nigt3* | 1.1770±0.0909 | 0.0377* |  |  |  | *nigt3* | 795.40±43.67 | 0.0907 |
|  |  | *nigt2/3-1* | 1.2540±0.1017 | 0.0012** |  |  |  | *nigt2/3-1* | 854.21±75.52 | 0.0034* |
|  |  | *nigt2/3-2* | 1.3053±0.1642 | 0.0010** |  |  |  | *nigt2/3-2* | 891.57±46.32 | 0.0001** |
| Shoots | 0.2 | WT | 0.0535±0.0785 |  |  | Shoots | 0.2 | WT | NA |  |
|  |  | *nigt2* | 0.5811±0.0836 | 0.1873 |  |  |  | *nigt2* | NA |  |
|  |  | *nigt3* | 0.5742±0.0753 | 0.2252 |  |  |  | *nigt3* | NA |  |
|  |  | *nigt2/3-1* | 0.6260±0.0784 | 0.0113* |  |  |  | *nigt2/3-1* | NA |  |
|  |  | *nigt2/3-2* | 0.6066±0.0711 | 0.0287* |  |  |  | *nigt2/3-2* | NA |  |
|  | 2 | WT | 2.3920±0.2527 |  |  |  | 2 | WT | 103.10±15.00 |  |
|  |  | *nigt2* | 2.5010±0.3198 | 0.3642 |  |  |  | *nigt2* | 111.46±15.50 | 0.1930 |
|  |  | *nigt3* | 2.5364±0.3374 | 0.2481 |  |  |  | *nigt3* | 110.71±12.12 | 0.1853 |
|  |  | *nigt2/3-1* | 2.8092±0.4478 | 0.0192* |  |  |  | *nigt2/3-1* | 124.23±14.46 | 0.0020** |
|  |  | *nigt2/3-2* | 2.7128±0.3602 | 0.0102* |  |  |  | *nigt2/3-2* | 120.67±8.822 | 0.0008** |
|  | 4 | WT | 2.5232±0.3276 |  |  |  | 4 | WT | 118.12±11.53 |  |
|  |  | *nigt2* | 2.7384±0.3448 | 0.1313 |  |  |  | *nigt2* | 129.62±11.94 | 0.0198* |
|  |  | *nigt3* | 2.7018±0.3802 | 0.1948 |  |  |  | *nigt3* | 129.8±13.62 | 0.0617 |
|  |  | *nigt2/3-1* | 2.8678±0.3457 | 0.0200* |  |  |  | *nigt2/3-1* | 140.82±13.79 | 0.0002** |
|  |  | *nigt2/3-2* | 2.9494±0.2483 | 0.0016* |  |  |  | *nigt2/3-2* | 143.10±21.11 | 0.0016** |

**Supplemental Sequence of promoters used in Y1H assay (The yellow highlight indicates B-box motif).**

>OsNRT1.1A

CTTACAAAACCAACATTTCACTTTTATTTAATTCATCCATTTATGTCCTTAAATAACTACAGTAAACTCAATTCCATCTATAACCCTATCATGTATGTATTTGGTTGAATAGGTAGAGTAGGTCAGGTTGGATCGAACCAATTTTCAAAATGTTTAGTTTGAAGATAGGTGGGATAAGGTTGATTCCGTAACGAGAATATTCCCCCGAAATCCGAGTTAGCTTCACACCTTAAAATCTAGCATATCAATCCAACACATTTACTCTCTATTATTGACATGTGGGCCATAACCTCCCATACCTAAAAAAGACAAAACCACTCCATCCCTCATGGCAAACTGATAATGGGACCACCATAAAAAAAACAAGGATAAGTCTAACTCATTTCACCTTATCTC

>OsNRT1.1B

CCACTATACGTTCAATGCGAGGTAGAAAGATGCAGGTTCAAATTGTTGTTGCTACCGACTAATTAATCTGTTCATCTGAATCTGAATTTTTGTCATATTTTGGGCCCTTCCAGTTTCAAATATATATCGATCGGGCAGTGGTTTTAACTGAACTAATGTACTTACGAGGATGATCAAAACAAAATTAATGATCTAAAAGCTGAAATGCATGAGAACCTAATTGTGAGGCTCCTAGTTACACGACAAATCAGAATCTGATGTAGTGAATATTTTTCTCCAAGCGGGTTAGTAGTATCTTTCAGATTCACACGCATCTCTTTTCCCTACAAACACTTCACCTTTTTTGCAAGATGCTACTACTAATTACTAAACATCACAGCCTCAGAGAATGGGCAGCACACATTGATCATACCACACACTGTTCCCTGCACATAAAAAGTTTCAGTCAAAAAATTATATATCTTGATACACAGTAGTCAAGATCACTTGTGTTCCAT

>OsNRT2.2

TTGTTGTTGATCCTGACACGCCGTTGCTTTGCTTCAGGCCGTCATGAAAAAAAACCAGTGCATATCTCATGGCAAGAAGAAGTGCAGGTTGTGAACTGAAACTGCAAACATGAAGAGAAAATACAGAAGTTTATGAAGAAGCTCCCATCAATCCAAGCAAAGTTTTTTTTTTCTTGTTATTTAATAGAACAAAGTTACAATGCCAGAGTAAACAGTGCTGAGCCACAATTCTTGGAATCTCATTTTACTCTGAACACTGAAGGATTCTGTGGACAGATACAGAACATAACATAACAGAACAGAACAGAAAAGGAAGAGAAATTAATTAATCGGCAAGCAACAGATCGATTCTGCGCACGCATCTGATGGGATATTCTGATCCTTGGTGGATCCCATGGGATAAAGGGCAGTCTTCTCCGGAGATGCGGCGCCGAGGTTATCTCCTTTATCAATTCATTTCAGCTTAATTTAAACGGTTCTTTGCTTTCCTG

>OsAMT1;2

TATAATTTATTTTATTATTAGTCTATATTTAATACTTCAAAATATGTATGCGTGTACTTCAAAAACTTTATATCAAAAAACTAAACACAGCCTCCAGGCCGCAGCCTACAGTAGGCCTATAGAGAGATTCCACGGGATTCGATGAACTACGACCACGAACAGGAGGGGGACAAATCAACAAGCAAATCATAGGGGTCCCACATTTCAGAGGTAGCCAAAGATTCACTGGCAGGTGGGCCCTTCACACTTTGAAGGAATCAACAACGACACCCCCCAAGTCATGGATTCCTTCTCGCTCCCTCTCCACGTCGCCTATAAATCCGACGCGGCCGCTCCCCACTCCACCCACAGCCCACACTTCCATTGCTCCTCCCCTCTCCTCTACAGTCTGTGTTGAGCGCGCGTCGAGGCGGCGAGGATGGCAACGTGCGCGGATA

>OsAMT1;3

TACATGCATGCCTGAAGAACTTAAACAGTTAATCAACAAAGTCAATGGATATTACGCATACAAGTATATGGTTGTATATATGCAACTTCATGACACAACAGTATGCGTATAGTCGGACGTGACGACAAGCAACTACCTCGTGCAAGGATGCGAGGAGATACCAGATTAAAACCTGTAATACTTGAAGTTGACAAAATGCGATTCTTCAGACGATATAATAACAAGAACATTTCTGAATCTTCCTTTAAAAAATGTTGAATGCATAAAAGAATCTTAGCTGTGATGGCAACAACACGACTTTCTGATAGTGACATTGGATCTTAGTTGAATCTGGCATCTTGCGTATGCGACCTTGCTTGGATCTGTCGGATACTCCCAACCCAGCATAAATTACTGATGTCTGAAACTTTCTGAGCAAAGCGGGAACTCAGGCTAGTTGAGTCGCTCATCATCAAAAGTCAAGACAATACTTAAGTAAAAACAAAACAAATATCACTGTCGCAAAACCAGTG

>OsAMT2;1

TTAGTATCTCCTGTTCGTGCAGGTAGTACATCGTATCTGCTCCGTGAGTTGGTTTCGTGCGCATCAATTACACTGTACTCCTTCTATAGTACAAGAGGAGATTCTTTTTTCAGAACAAGCTCAAATTCTAATACAAGTAGGATGATACCCAAGGTCCGATACAAGGAGGGTGGCGATACTGCTGAATCTACATGTCTATTCTCCACAAAGAAGCCAATCAAGGTTTGGACTTAGGAATCTCCTCAACTACCATCATTAAAAAGACAAAGTGGGCATAAAGAACACGAAAAACTCAAGTCATGTATCAGGCCTATTTATTTTCTTTTCCTTCAGGACAAACCAAGACATGCCACATCTATGAAGAGAAGAAAAGTTTTAAGTAAAAACTAAGGAAGAAAAGGCGCATCACGCCACA

>OsSPX4

CGACTAGCCACTAGATCTAGGGGTCGGACGGCTAGGCAGTGGGGATAGGATTAATATGATTTTTTAAAAAACAATTTTCCTATAGACTTTTTTTATAAAAAAATACACCGTTTAACTATATGGAAACCGTGCGTACGAAAAACGGTTTCGTTTTGAAACTTATGACATGTTTCATTAAAAATGTGATAAATCGAGATAACAACTAATTTGCTCTATAAAAACCACACAAAAAAAAGGAATCTTTTTAGGCGGATGGAATCAATTCTACTCCTGTTCTTTTACTTCTGTACAGAAACCGGTAACTGGAAGAAGTAAAACCCAGGCAAGGCAGAGAGCCAGCGAAAGCGGCAAAAGCATCGCATCATCGCAGCCGCCGAGGGGAAGAAGAAGAAGAAGACAAGGGACCCACGAGGGGAATATCCCACATCCGGGCCCACCTGACAGTGACCCAACA

>OsNIGT1

TTGCCAAGGCCCCAAGCCCCCAACCCCTACAGGGTGTCGTGTCAGCCACCCCCGCGGCCCCGCCCAGGTGGCCCCCGTTCCAGATTCCGCATCGCTCCCTTCGATCCGCGGCATCCTCCGCTCCCCTCGTCCAAGTCGCCTTCTTCCTTATCCAATCCCCTACCTGCGCACTCTCCAGACTCGCCCAGTAAACAAAAACAAGTGGGTCCCACATCCGAGCCGAAGGAACGGAATCCCCCTCCGAATATTCACCACCACCACCCTCCTTTTACTTATTTCCGCCCGTCCCAATCCAGCCACCCGCTCGCCCGTCCGTTCGATTCGACGACACCTTCATTTTTATTTTCTTCCTTGTGTAATAAGAAGACGACTAGTGGTAACAAAAAGATTCGCTCCCTACGCGACGCCCCGACCCCGATTCCCTTCGCTTCCTACTCCTTCCTCTTCCTTGTTCCTTCCTTCCTTCCTCA

>NIGT2

CATCACACAATTTTGAATTCCAGAAAGCACAAAATGCTATAGCAAATGCGACAAAAAGATAGACAAGAAGTTAAACAAGCGACAAATCAAAGGAGAAGTGCACAAATGTCACTGCAGGTGCTCGACGAATTAAAGGCGTGTGCTGTTACCGAATCATTCGAAGCAAGCCAAAAAGAAAAAAATAATAATTATGTTATTTTGTACGCATATACGAACAACTCATCGCTTCAAAGAGAAGCAATCGAAAAGCGCAAGAATTAAAAACCGATATCTTCTAGATATCGGTTTTTAATAGCATCATATCCGTCCGTTCTATCCTACGCATATCTTTACCTCTAGCAATTAATCCGTCCCAAATCTGAATATCTTCAATCAATTTCTCGCCCACAGATTAATTACACCAGCCGAGTAGCCGACCCTCACCACGGAGATATTTCAGTTTCCATGTCACAACCCCATAAAACCATCAGAATTACAACATCTACATAAAAAAAACTAACTTTATAAATCAGTATCAT

>NIGT3

GCACCCCCCACAAAAACCCAAAATCTACTGCACCATCCGGCTTTGGAGCGTTGCATGCAGCCGTCAAAAATCCCCTCCTCTCAAAAACCAATCGCCCCAACCGATGGGCAGGCTGCGCCTACGCTTCGCCGCGAGACCAGGGAATCGAAGCCGCAGGGGGAAGAATCCACGGAGGATTATTCCAACACACACGTAACGCAAGCTTGCCATTATCTAAAAACGCAAGCCACCAAATACTCGTACTACTACAAAACGAAGCTACGACACGCAAAGGACCGCATGGTAAATAAAACTATTCAACCTCCAGG

>NIGT3

AAGTACGAGCGTGTCATGGTTGAAATTTTGTGAGATTCATAGAAGCTTGTGGTAAGGATATGCACGCGTACGTGGTGATTTGTCATGTAAAGCCTCCTGTGAATGAGCGATAAGCAAAACATATTTTGTACAGCCGATTCGGATCCTTCGGGAATACACGGAATCATTTTCCACCGTGCCGGACAAGCTGAACACTGACAGGTCTTTGGACCATTGCATGCAGACACGCACCCCCCACAAAAACCCAAAATCTACTGCACCATCCGGCTTTGGAGCGTTGCATGCAGCCGTCAAAAATCCCCTCCTCTCAAAAACCAATCGCCCC
